# Structural Snapshots for Mechanism‐Based Inactivation of a Glycoside Hydrolase by Cyclopropyl Carbasugars

**DOI:** 10.1002/anie.201607431

**Published:** 2016-10-26

**Authors:** Christopher Adamson, Robert J. Pengelly, Saeideh Shamsi Kazem Abadi, Saswati Chakladar, Jason Draper, Robert Britton, Tracey M. Gloster, Andrew J. Bennet

**Affiliations:** ^1^ Department of ChemistrySimon Fraser UniversityBurnabyBritish ColumbiaCanada; ^2^ Biomedical Sciences Research ComplexUniversity of St AndrewsNorth HaughSt Andrews, FifeUK; ^3^ Department of Molecular Biology and BiochemistrySimon Fraser UniversityBurnabyBritish ColumbiaCanada

**Keywords:** carbocycles, enzyme mechanisms, glycoside hydrolases, inhibitors, X-ray crystallography

## Abstract

Glycoside hydrolases (GHs) have attracted considerable attention as targets for therapeutic agents, and thus mechanism‐based inhibitors are of great interest. We report the first structural analysis of a carbocyclic mechanism‐based GH inactivator, the results of which show that the two Michaelis complexes are in ^2^H_3_ conformations. We also report the synthesis and reactivity of a fluorinated analogue and the structure of its covalently linked intermediate (flattened ^2^H_3_ half‐chair). We conclude that these inactivator reactions mainly involve motion of the pseudo‐anomeric carbon atom, knowledge that should stimulate the design of new transition‐state analogues for use as chemical biology tools.

Life is supported by a myriad of enzyme‐catalyzed reactions; one such life‐sustaining activity is the transfer of carbohydrate groups from one biomolecule to another.^1^ Understanding how these fundamentally important transfer reactions occur in nature guides researchers in the design of compounds (inhibitors/activators) that modulate the activity of these biological catalysts. Glycoside hydrolases (GHs or glycosidases) are a type of carbohydrate‐processing enzyme used in the reshaping of biomolecules.^2^ Most GHs catalyze glycoside hydrolysis through one of two distinct processes that are reliant on a pair of active‐site aspartic and/or glutamic acid residues. Hydrolysis by such retaining glycosidases involves two sequential inversions of configuration at the anomeric center, the first of which results in the formation of a covalent glycosyl‐enzyme intermediate (Figure 1 a). In contrast, inverting glycosidases operate via a single inversion of configuration at the anomeric center. In both cases, pyranosylium ion like transition states (TSs), which can have half‐chair (^4^H_3_/^3^H_4_), boat (^2,5^B/B_2,5_), or envelope (^4^E and ^3^E) conformations (Figure 1 b),^2^, ^3^ are implicated. By exploiting this knowledge, we recently reported that the cyclopropyl‐containing carbasugar **1** is a mechanism‐based inactivator of an α‐d‐galactosidase from *Thermotoga maritima* (*Tm*GalA; Figure 1 c).^4^ Within the enzymatic active site, **1** likely forms a transient bicyclobutenium ion (**1^+^**), and enzyme inactivation occurs through alkylation of the catalytic nucleophile Asp 327.

**Figure 1:**
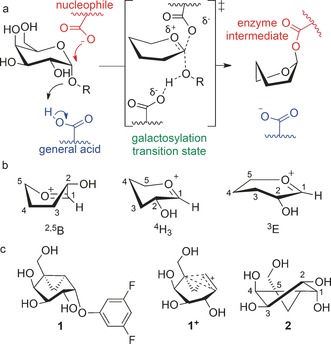
a) Mechanism of galactosylation for a retaining α‐galactosidase; for clarity, hydroxy groups are not shown for the transition state or intermediate. b) Representative conformations for pyranosylium ions (^2,5^B, ^4^H_3_ and ^3^E); only the C2 hydroxy group is shown for clarity. c) Structures of mechanism‐based inactivator **1**, presumed bicyclobutenium ion intermediate **1^+^**, and hydrolyzed inhibitor **2**, which is drawn in a ^2^H_3_ conformation.

Given the current desire for small‐molecule transition‐state analogues (TSAs) as leads for therapeutic development,^5^ it is important to understand how GHs stabilize cationic TSs.^4^ Of note, GHs are among the most catalytically proficient enzymes, since they accelerate hydrolysis of glycosidic bonds by up to 10^17^‐fold.^6^ Therefore, an understanding of the distinct ring conformations of the substrate and product Michaelis complexes and the covalent adduct^7^, ^8^, ^9^ is critical to the design of effective TSA inhibitors.

Herein, we describe the first conformational itinerary for a non‐pyranosyl mechanism‐based inactivator of a glycoside hydrolase. That is, we have structurally characterized the Michaelis complexes of bicyclic carbasugar **1** and the resulting hydrolyzed product **2** with a GH36 α‐galactosidase (*Tm*GalA). We also present the de novo synthesis of a fluorinated inactivator (**3**) and structural characterization of its covalent adduct with *Tm*GalA.

To gain insight into the conformational itinerary displayed by **1** upon binding to *Tm*GalA, we undertook structural studies using X‐ray crystallography. Although a structure of *Tm*GalA has been reported (PDB ID: 1ZY9), in our hands, *Tm*GalA crystallized under different conditions and in a different space group to that reported (see the Supporting Information). The apo *Tm*GalA structure was solved to 1.80 Å resolution (PDB ID: 5M0X) by using molecular replacement with the structure of *Tm*GalA (PDB ID: 1ZY9) as the search model. Like other GH36 enzymes,^10^ *Tm*GalA comprises three domains; an N‐terminal β‐sandwich fold domain, a canonical (β/α)_8_‐fold domain housing the active site, and a C‐terminal β‐sandwich domain with a Greek‐key motif (Figure 2 a).

**Figure 2:**
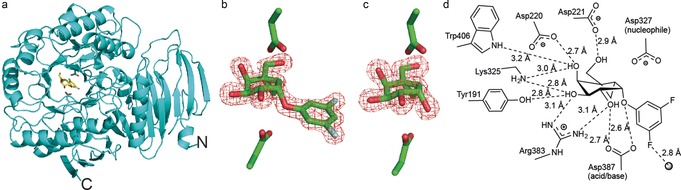
Structural analysis of *Tm*GalA following crystal soaks with the carbasugar inactivator **1**. a) A ribbon representation of *Tm*GalA (with “N” and “C” denoting the N and C termini, respectively) in complex with **1** (shown as a yellow stick model). The catalytic residues (Asp327 and Asp387) are shown as cyan stick representations. b, c) Stick representations of (b) *Tm*GalA in complex with **1** (PDB ID: 5M12) and (c) *Tm*GalA in complex with **2** (PDB ID: 5M16). In each case, the observed electron density for the maximum likelihood weighted 2 *F*_obs_−*F*_calc_ map is contoured at 1.5σ, and the catalytic nucleophile (Asp327) is shown above the pseudo‐sugar, with the catalytic acid/base (Asp387) is shown below. d) Schematic depiction of the interactions between *Tm*GalA and **1**.

Given the short half‐life (<1 min) for reaction of the enzyme–inhibitor complex (E:I complex) between **1** and *Tm*GalA in solution,^4^ apo *Tm*GalA crystals were soaked briefly (5 s to 30 min) with **1** in an effort to observe the covalent complex formed through nucleophilic trapping of the bicyclobutenium ion intermediate (Figure 1 c; **1^+^**) by Asp 327. Surprisingly, these crystals failed to yield a structure with any evidence of a small molecule bound in the active site of *Tm*GalA. Crystals soaked for longer periods (1 h to 7 days) did, however, lead to structures with electron density present in the active site. Structures for *Tm*GalA in complex with intact **1** (from crystals soaked for 1 h; Figure 2 a, b) with data to 1.53 Å resolution and of *Tm*GalA in complex with the hydrolyzed cyclopropyl carbasugar **2** (from crystals soaked for 7 day; Figure 2 c) with data to 1.62 Å resolution were obtained. Together, these structures provide unique insight into the conformational itinerary of the carbasugar through stages mimicking both the reactant and product Michaelis complexes.

The structure of *Tm*GalA in complex with **1** provides a rare glimpse of the interactions within a Michaelis complex, which is formed fleetingly upon substrate binding prior to catalysis. Here, the carbasugar binds in a half‐chair ^2^ *H*_3_ conformation (Figure 2 b). A hydrogen bond is evident between the glycosidic oxygen of **1** and Asp387, the acid/base residue that is primed to donate a proton to aid departure of the leaving group. In addition, the C2‐OH hydrogen bonds with Asp387 and Arg383, C3‐OH hydrogen bonds with Arg383, Tyr191, and Lys325, C4‐OH interacts with Lys325, Asp220, and weakly with Trp257, and C6‐OH hydrogen bonds with Asp221 (Figure 2 d). There are also hydrophobic interactions formed between **1** and Trp190 and Trp257. One of the two fluorine atoms on the aromatic leaving group interacts weakly with a water molecule. Notably, the structure of *Tm*GalA in complex with **2** shows that the inactivator has been hydrolyzed with retention of configuration. This new finding shows that within the confines of the enzymatic active site, the cyclopropyl ring structure is maintained, in contrast to the reactions of such systems in solution.^11^ The hydrolyzed carbasugar **2** binds in an identical position and conformation to that observed for the complex with **1**, and the interactions with active‐site residues are also the same. Indeed, the pseudo‐anomeric C1‐OH hydrogen bonds with the catalytic acid/base residue (Asp387) and a water molecule (which was not present in the complex with **1**) that partially fills the void created by the departure of the aromatic leaving group.

Considering the greatly extended half‐life for reaction of the E:I Michaelis complex, it follows that the inactivator **1** undergoes a pseudo‐glycosylation reaction within the crystalline phase via a transition state that has a different activation free energy barrier to that of the equivalent reaction in aqueous solution.^4^ Also, hydrolysis of the Asp327‐linked carbasugar intermediate, which has only been detected previously by mass spectrometry,^4^ proceeds via a perturbed TS free energy pathway in the solid state. These two observations inspired us to design a second‐generation inactivator with a significantly lower reactivation rate, which would enable structural analysis of the covalently bound complex with *Tm*GalA in the solid state. Toward this goal, we elected to adopt a common and very successful strategy used in the design of pyran‐based GH inactivators: improving the leaving‐group ability of the pseudo‐aglycone to ensure rapid formation of the covalent enzyme–inhibitor complex while replacing the C2 hydroxy group with a fluorine atom to slow the subsequent hydrolysis.^12^ Accordingly, we designed compound **3**, in which the pseudo‐C2 hydroxy group is replaced with fluorine atom and the leaving group is a 2,4‐dinitrophenylate rather than a 3,5‐difluorophenoxide anion (Scheme 1).

**Scheme 1:**
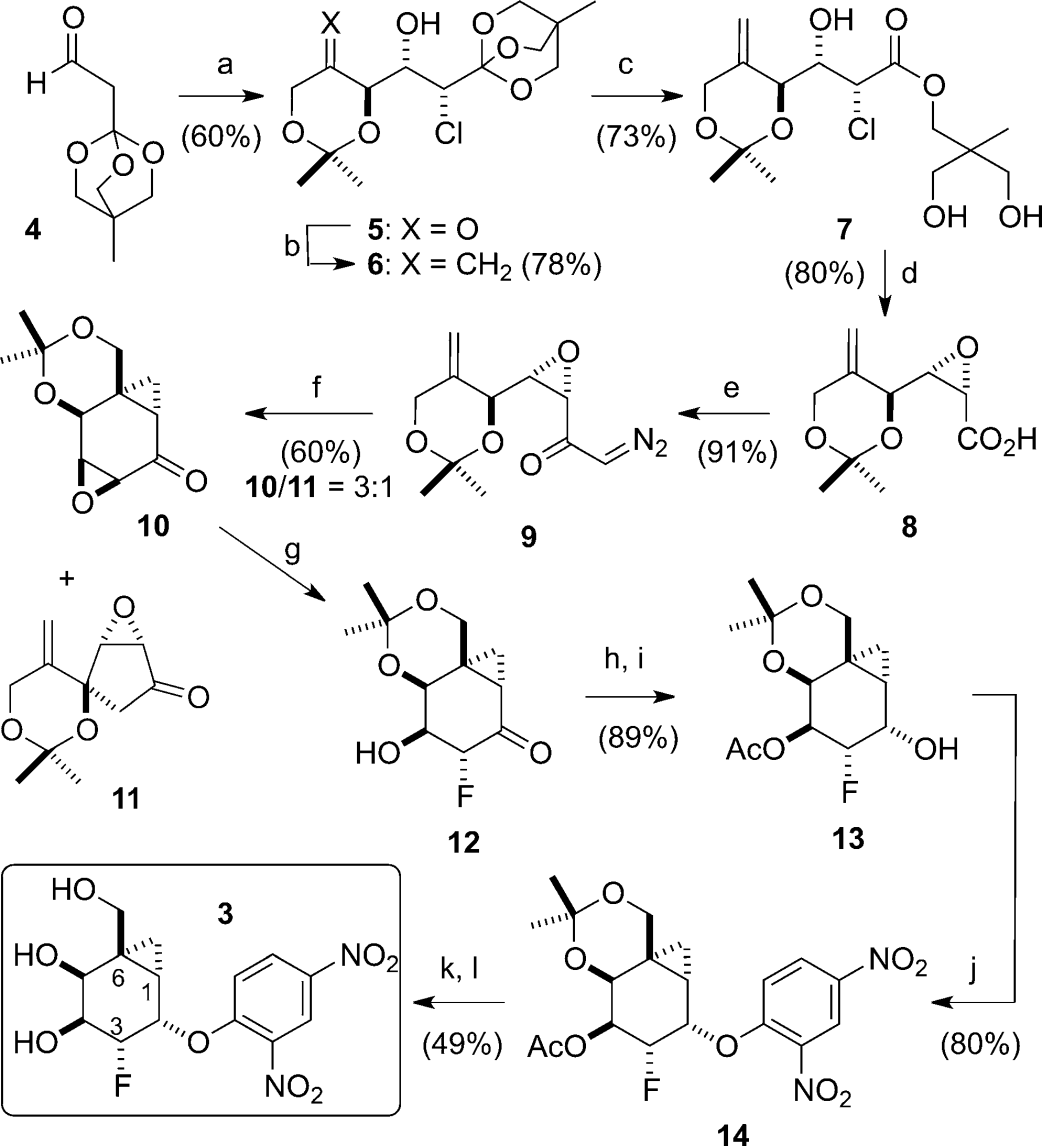
Synthesis of inactivator **3**. a) 2,2‐dimethyl‐1,3‐dioxan‐5‐one, NCS, (*R*)‐proline (80 mol %), CH_2_Cl_2_; b) 5‐(methylsulfonyl)‐1‐phenyl‐1*H*‐tetrazole, LiHMDS, THF, −78 °C, then **5**; c) PPTS, dioxane; d) NaOH, THF, H_2_O, 50 °C; e) Et_3_N, isopropyl chloroformate, THF, −15 °C to 0 °C then TMSCHN_2_, MeCN, RT; f) Rh_2_(OAc)_4_ (2 mol %), 4 Å mol sieves, CH_2_Cl_2_, RT; g) TBAF, THF, 0 °C (30 % from **9**); h) Ac_2_O, DMAP (cat), pyridine, CH_2_Cl_2_, RT; i) L‐selectride, THF, −78 °C; j) quinuclidine, 4 Å mol sieves, 2,4‐dinitrofluorobenzene, DMF, RT, 24 h; k) K_2_CO_3_, MeOH, 0 °C, 0.5 h; l) Amberlite^®^ IR 120 H^+^ resin, MeOH, RT, 24 h. LiHMDS=lithium bis(trimethylsilyl)amide; NCS=*N*‐chlorosuccinimide; PPTS=pyridinium *p*‐toluenesulfonate; TBAF=tetrabutylammonium fluoride; DMAP=4‐dimethylaminopyridine.

Our concise de novo synthesis of the densely functionalized fluorocarbasugar **3** is depicted in Scheme 1, and began with the coupling of orthoester **4** 13 with 2,2‐dimethyl‐1,3‐dioxan‐5‐one using our one‐pot organocatalytic α‐chlorination/dynamic kinetic asymmetric aldol reaction.^14^ Notably, this convenient reaction provided the β‐ketochlorohydrin **5** (>96 % *ee*) as a single diastereomer in analytically pure form through precipitation and thus could be executed on a greater than 10 g scale.^15^ After screening several olefination methods, we found that Julia–Kocienski olefination using methylsulfonyl phenyltetrazolyl^16^ afforded the alkene **6** in excellent yield. A two‐step hydrolysis of the orthoester then gave the unstable epoxy acid **8**, which was immediately coupled to TMS‐diazomethane to afford the diazoketone **9**. As a key design element, we envisaged coincident formation of the bicyclo[4.1.0]heptane core and the all‐carbon quaternary center at C6 through an intramolecular rhodium‐carbenoid cycloaddition reaction.^17^ In contemplating the stereochemical outcome of this annulation event (**9**→**10**), it was expected that the transition‐state structure leading to the l‐*altro* cyclopropane (not shown) would involve considerable strain and, consequently, that this process should favor formation of the desired d‐*galacto*‐configured **10**. We were delighted then that our initial attempts to effect the intramolecular cycloaddition^17^ by using Rh_2_(OAc)_4_ in anhydrous^18^ CHCl_3_ delivered a mixture of the desired cyclopropane **10** and the unexpected C−H insertion product **11** (**10**/**11**=2:1). While reaction in benzene afforded a 1:1 mixture of cyclopropane **10**/**11**, in CH_2_Cl_2_, an acceptable 3:1 mixture of these compounds was produced in around 60 % yield.^19^ Unsurprisingly, the strained tricycle **10** proved to be labile and partially decomposed during chromatographic purification. Therefore, it was optimal to treat the crude cyclopropanation reaction product directly with fluoride and isolate the stable α‐fluoroketone **12**. Acetylation followed by reduction with l‐Selectride afforded the fluorohydrin **13**, which underwent nucleophilic aromatic substitution with dinitrofluorobenzene to afford the arylether **14**. Finally, sequential removal of the acetate and acetonide protecting groups delivered the second‐generation inactivator **3** in good overall yield.

Despite the enhanced leaving‐group ability of the dinitrophenolate in carbasugar **3**, this compound proved to be significantly less reactive with *Tm*GalA than inactivator **1**. Specifically, at both 25 and 37 °C, we noted little or no enzyme inactivation over 6 h. After raising the temperature to 50 °C and the concentration of **3** to 500 μm, *Tm*GalA activity decreased with a first‐order rate constant of 2.86×10^−5^ s^−1^. We therefore measured the kinetic parameters for inactivation of this thermostable enzyme by **3** at 60 °C and found *k*_inact_/*K*_i_=0.71±0.19 m ^−1^ s^−1^ and *k*_inact_=(2.25±0.21)×10^−4^ s^−1^ (Figure 3). For comparison, the inactivation parameters for **1** with *Tm*GalA at 37 °C are *k*_inact_/*K*_i_=160±40 m ^−1^ s^−1^ and *k*_inact_=(1.42±0.11)×10^−2^ s^−1^.^4^ We also attempted to measure the rate constants for reactivation of the modified *Tm*GalA after removal of excess **3**, however, after 5 days at 60 °C, we observed no increase in enzyme activity,^20^ thus suggesting that hydrolysis of the covalent adduct is exceedingly slow.

**Figure 3:**
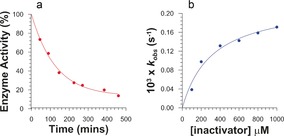
Reaction kinetics for the inactivation of *Tm*GalA by compound **3**. a) A typical plot of enzyme activity versus incubation time with [**3**]=400 μm. b) A plot of the first‐order rate constant for loss of *Tm*GalA activity as a function of inactivator concentration. Conditions for all experiments are *T=*60 °C in 50 mm HEPES buffer, pH 7.4.

Using the information gleaned from the kinetic analysis of *Tm*GalA with **3**, we incubated *Tm*GalA with **3** at 60 °C overnight prior to crystallization, which was followed by buffer exchange to remove any unreacted **3** and the 2,4‐dinitrophenol produced during inactivation. New crystallization conditions were identified (see the Supporting Information), and we obtained structural data for this modified *Tm*GalA to 1.55 Å resolution. As indicated in Figure 4 a, the electron density in the active site of *Tm*GalA unambiguously confirms that the carbasugar skeleton was covalently linked to the nucleophilic acid (Asp327) of *Tm*GalA.

**Figure 4:**
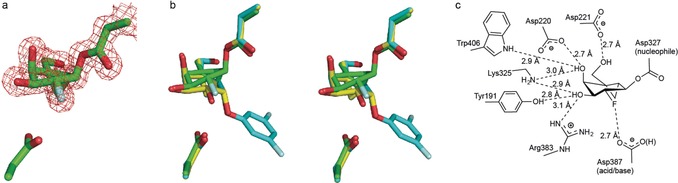
Structural analysis of *Tm*GalA following co‐crystallization with the carbasugar inactivator **3** (PBD ID: 5M1I). a) Stick representation of *Tm*GalA covalently labeled by **3** on the catalytic nucleophile (Asp327). The observed electron density for the maximum likelihood weighted 2 *F*_obs_−*F*_calc_ map is contoured at 1.5σ. The catalytic acid/base (Asp387) is shown below the pseudo‐sugar. b) A divergent stereo stick representation of the overlap of *Tm*GalA in complex with **1** (cyan) or **2** (yellow) or labeled by **3** (green) c) Schematic depiction of the interactions between *Tm*GalA and **3**.

The structure of *Tm*GalA alkylated by the carbocyclic fragment from **3** provides insight into the reactivity of the nonclassical carbocation intermediate formed within the constraints of the active site during catalysis. Interestingly, the trapped carbasugar intermediate maintains the bicyclo[4.1.0]heptane framework of **3**, rather than one of the many possible rearranged skeletons,^11^, ^21^ with an inverted stereochemistry at the pseudo‐anomeric center. Thus, the reaction appears to proceed via a simple S_N_2 displacement of the dinitrophenolate. The carbasugar binds in a non‐perfect half‐chair ^2^H_3_ conformation, where the C2 atom is also slightly above the plane containing the two bridgehead carbons and C1 and C4.^8^ Despite the fairly large movement of the carbasugar to accommodate the covalent bond to Asp327 when compared to the complexes with **1** and **2** (the C1 atom moves by 1.56 Å), the positions of the three unaltered hydroxy groups are essentially unchanged (Figure 4 b). That is, the majority of the active‐site interactions are unchanged and additionally, the 2‐fluoro group displays clear interactions with the catalytic acid/base residue Asp387 (Figure 4 c).

In summary, we have described for the first time the conformational itinerary that occurs during the inactivation and reactivation cycle for a glycoside hydrolase reacting with a carbasugar mechanism‐based inactivator. Given that enzyme dynamics is an important component to catalysis,5b our findings provide a basis for designing TSA inhibitors that incorporate the features of the bicyclobutenium ion intermediate resulting from the reaction of these novel bicyclo[4.1.0]heptane inactivators.

## Supporting information

As a service to our authors and readers, this journal provides supporting information supplied by the authors. Such materials are peer reviewed and may be re‐organized for online delivery, but are not copy‐edited or typeset. Technical support issues arising from supporting information (other than missing files) should be addressed to the authors.

Supplementary
