# Supplementary material for: Structural Snapshots for Mechanism‐Based Inactivation of a Glycoside Hydrolase by Cyclopropyl Carbasugars
Source: Angew Chem Int Ed Engl. 2016 Oct 26;55(48):14978–82. doi: 10.1002/anie.201607431 (PMC5132143; doi:10.1002/anie.201607431)
Supplement: Supplementary file 1 — Supplementary [file ANIE-55-14978-s001.pdf]

Angewandte Chemie
Eine Zeitschrift der Gesellschaft Deutscher Chemiker

Supporting Information

Structural Snapshots for Mechanism-Based Inactivation of a Glycoside Hydrolase by Cyclopropyl Carbasugars

Christopher Adamson, Robert J. Pengelly, Saeideh Shamsi Kazem Abadi, Saswati Chakladar, Jason Draper, Robert Britton, Tracey M. Gloster,* and Andrew J. Bennet**

anie_201607431_sm_miscellaneous_information.pdf

Supporting Information

Table of Contents

General considerations SI 2

Synthetic experimental procedures SI 3

Protein expression, purification, crystallization and kinetics SI 10

Synthetic scheme SI 12

X-ray crystal structure data summary SI 13

References SI 14

NMR spectra for all new compounds SI 15

General considerations

All anhydrous reactions described were performed under an atmosphere of nitrogen using flame-dried glassware. Normal phase column chromatography was carried out with 230-400 mesh silica gel (Silicycle, SiliaFlash® P60) following the technique described by Still.[1] Reversed phase column chromatography was carried out using a CombiFlash® Rf+ unit equipped with a 43 g RediSep® Rf reversed phase C18 cartridge. Concentration and removal of trace solvents was done with a Büchi rotary evaporator using a dry ice/acetone condenser and vacuum applied from a Büchi V-500 pump.

All reagents and starting materials were purchased from Sigma Aldrich, Alfa Aesar, TCI America or Arcos and were used without further purification. All solvents were purchased from Sigma Aldrich, EMD, Anachemia, Caledon, Fisher or ACP and used without further purification unless otherwise specified. CH_2Cl_2 was freshly distilled over CaH_2; THF was freshly distilled over Na metal/benzophenone. Cold temperatures were maintained by use of the following conditions: 0 °C, ice-water bath; −78 °C, acetone-dry ice bath; temperatures between −78 °C and 0 °C required for longer reaction times were maintained with a Neslab Cryocool Immersion Cooler (CC-100 II) in a 2-propanol bath.

Nuclear magnetic resonance (NMR) spectra were recorded using $CDCl_3$ or CD_3CN. Signal positions (δ) are given in parts per million from tetramethylsilane (δ 0) and were measured relative to the signal of the solvent (^{1}H NMR: $CDCl_3$: δ 7.26, CD_3CN: δ 1.94; ^{13}C NMR: $CDCl_3$: δ 77.16, CD_3CN: δ 118.26). Coupling constants (*J* values) are given in Hertz (Hz) and are reported to the nearest 0.1 Hz. ^{1}H NMR spectral data are tabulated in the order: multiplicity (s, singlet; d, doublet; t, triplet; q, quartet; m, multiplet; br., broad), coupling constants, number of protons. NMR spectra were recorded on a Bruker Avance 600 equipped with a QNP or TCI cryoprobe (600 MHz), Bruker 500 (500 MHz), or Bruker 400 (400 MHz). Infrared (IR) spectra were recorded on a Perkin Elmer Spectrum Two™ Fourier transform spectrometer with neat samples. Only selected, characteristic absorption data are provided for each compound. High resolution mass spectra were performed on an Agilent 6210 TOF LC/MS using ESI-MS or were carried out by the Notre Dame University Mass Spectrometry Department using EI technique. Optical rotation was measured on a Perkin Elmer 341 Polarimeter at 589 nm.

Experimental procedures

Ethyl ((3-methyloxetan-3-yl)methyl) propanedioate (**S1**)

Diethyl malonate (450 mL, 2.96 mol) and (3-methyloxetan-3-yl)methanol (60.0 g, 0.59 mol) were stirred for 48 hours at 150 °C in a vessel equipped with a reflux condenser. The mixture was then allowed to cool to room temperature, and the diethyl malonate was removed by short-path distillation to afford **S1** (120 g, 555 mmol, 95%) as a colourless oil. This material (~85% pure by ^{1}H NMR) was advanced directly to the next step without further purification. To obtain an analytical sample for characterization, a small portion of the crude material was purified by column chromatography (2:1 / hexanes:EtOAc). ^{1}H NMR (400 MHz, $CDCl_3$) δ: 4.52 (d, *J* = 6.1 Hz, 2H), 4.38 (d, *J* = 6.1 Hz, 2H), 4.25 (s, 2H), 4.20 (q, *J* = 7.2 Hz, 2H), 3.42 (s, 2H), 1.34 (s, 3H), 1.28 (t, *J* = 7.2 Hz, 3H); ^{13}C NMR (101 MHz, $CDCl_3$) δ: 166.8, 166.5, 79.5, 77.4, 69.7, 61.8, 41.7, 39.2, 21.2, 14.2; HRMS: m/z calcd. for $C_{10}H_{16}O_5Na^+$: 239.0890 (M+Na); found: 239.0892 (M+Na); IR: 2966, 2874, 1729, 1144 cm^{-1}.

Ethyl 2-(4-methyl-2,6,7-trioxabicyclo[2.2.2]octan-1-yl)acetate (**S2**)

To a cooled (–15 °C) solution of diester **S1** (60.0 g, 250 mmol) in 500 mL CH_2Cl_2 was added $BF_3{\cdot}OEt_2$ (3.1 mL, 25 mmol). The mixture was stirred for 20 hours at –15 °C and then quenched by the dropwise addition of 17.5 mL Et_3N. After warming to room temperature, the mixture was concentrated and treated with 300 mL Et_2O, filtered through Celite®, and concentrated. Purification of the crude material by chromatography on a short column of neutral alumina (CH_2Cl_2) followed by recrystallization (hexanes/EtOAc) afforded **S2** (36.5 g, 169 mmol, 68%) as a white powder. m.p. = 53–56 °C (hexanes/EtOAc); ^{1}H NMR (400 MHz, CD_3CN) δ: 4.06 (q, *J* = 7.1 Hz, 2H), 3.87 (s, 6H), 2.60 (s, 2H), 1.19 (t, *J* = 7.1 Hz, 3H), 0.76 (s, 3H); ^{13}C NMR (126 MHz, CD_3CN) δ: 168.2, 107.8, 73.2, 61.1, 43.6, 30.9, 14.4, 14.2; HRMS: m/z calcd. for $C_{10}H_{17}O_5^+$: 217.1071 (M+H); found: 217.1065 (M+H); IR: 2879, 1738, 1180, 1047 cm^{-1}.

Compound **4**:

To a cooled (–78 °C) solution of orthoester **S2** (8.15 g, 37.7 mmol) in 370 mL CH_2Cl_2 was added DIBAL-H (1.0 M in hexanes, 49 mL, 49 mmol) dropwise under vigorous stirring. The reaction was then quenched by the dropwise addition of 2 mL MeOH and allowed to warm to 0 °C. After treatment with 1.5 mL H_2O, 1.5 mL 15% NaOH, and 4 mL H_2O, the cooling bath was removed

and the mixture was stirred for 15 minutes. The mixture was then dried (Na_2SO_4), filtered through Celite®, and concentrated to afford **4** (5.99 g, 35 mmol, 92%) as a colourless oil. This material (~90% pure by ^{1}H NMR) was advanced directly to the next step without further purification. To obtain an analytical sample for characterization, a small portion of the crude material was purified by column chromatography (2:1 v/v hexanes:EtOAc).

^{1}H NMR (400 MHz, $CDCl_3$) δ: 9.70 (t, *J* = 2.8 Hz, 1H), 3.94 (s, 1H), 2.65 (d, *J* = 2.8 Hz, 2H), 0.82 (s, 1H); ^{13}C NMR (101 MHz, $CDCl_3$) δ: 198.9, 107.0, 72.8, 49.8, 30.7, 14.6; HRMS: m/z calcd for $C_8H_{13}O_4^+$: 173.0808 (M+H); found: 173.0829 (M+H); IR: 2919, 1684, 1266 cm^{-1}.

Compound **5**:

To a solution of **4** (11.33 g, 65.8 mmol) in 330 mL CH_2Cl_2 at was added (*R*)-proline (6.06 g, 52.6 mmol), *N*-chlorosuccinimide (7.03 g, 52.6 mmol), and 2,2-dimethyl-1,3-dioxan-5-one (7.1 mL, 59 mmol). The mixture was stirred for 24 hours at ambient temperature, diluted with Et_2O (500 mL), washed with water (3 × 150 mL) and brine (150 mL), dried (Na_2SO_4), filtered, and concentrated. Purification of the crude material by trituration with Et_2O afforded **5** (9.60 g, 29 mmol, 43%) as an off-white powder. m.p. = 144–148 °C (Et_2O); α_D (CH_3CN, c = 0.67): +111°.

^{1}H NMR (400 MHz, CD_3CN) δ: 4.33 (ddd, *J* = 8.5, 2.7, 1.6 Hz, 1H), 4.27 (dd, *J* = 17.6, 1.3 Hz, 1H), 4.17 (dd, *J* = 8.5, 1.3 Hz, 1H), 4.11 (d, *J* = 1.6 Hz, 1H), 4.04 (d, *J* = 17.6 Hz, 1H), 3.95 (s, 6H), 3.40 (d, *J* = 2.7 Hz, 1H), 1.44 (*J* = 0.6 Hz, 3H), 1.37 (d, *J* = 0.6 Hz, 3H), 0.80 (s, 3H); ^{13}C NMR (101 MHz, CD_3CN) δ: 208.00, 109.1, 102.4, 73.3, 68.1, 67.7, 61.3, 31.3, 24.1, 23.7, 13.9; HRMS: m/z calcd. for $C_{14}H_{22}ClO_7^+$: 337.1049 (M+H); found: 337.1054 (M+H); IR: 3516, 2884, 1750, 1224, 1082, 1050 cm^{-1}.

Compound **6**:

To a cooled (–78 °C) solution of 5-(methanesulfonyl)-1-phenyl-1H-tetrazole (7.42 g, 33.4 mmol) in 50 mL THF was added dropwise a freshly prepared solution of 34.9 mmol of LiHMDS in 50 mL THF. The resulting pale yellow mixture was stirred for 30 minutes at –78 °C. A solution of **5** (5.00 g, 14.8 mmol) in 50 mL THF was then added dropwise and the mixture was stirred for a further 15 minutes at –78 °C. The mixture was then poured into EtOAc (1 L) and washed with H_2O (500 mL) and brine (500 mL), dried (Na_2SO_4), filtered, and concentrated. Partial purification of the crude material by column chromatography (30:70:1 to 50:50:1 v/v/v EtOAc:pentane:Et_3N) afforded a mixture of **6** and 5-(methaneslfonyl)-1-phenyl-1H-tetrazole

(7.59 g, estimated yield by ^{1}H NMR 3.92 g, 11.7 mmol, 79%) which was advanced directly to the next step without further purification. To obtain an analytical sample for characterization, a small portion of the crude material was purified by column chromatography (30:70:1 v/v/v EtOAc:pentane:Et_3N). α_D (CH_3CN, c = 0.62): +25.9°; ^{1}H NMR (400 MHz, CD_3CN) δ: 5.29 (m, 1H), 4.97 (m, 1H), 4.34 – 4.27 (m, 2H), 4.23 – 4.15 (m, 3H), 3.96 (s, 6H), 3.40 (d, *J* = 2.2 Hz, 1H), 1.43 (d, *J* = 0.6 Hz, 3H) 1.28 (d, *J* = 0.6 Hz, 3H), 0.81 (s, 3H); ^{13}C NMR (101 MHz, CD_3CN) δ: 144.0, 109.4, 109.3, 100.3, 73.6, 71.0, 70.5, 65.3, 61.2, 31.3, 28.0, 21.7, 13.9; HRMS: m/z calcd. for $C_{15}H_{23}ClO_6Na^+$: 367.1075 (M+Na); found: 357.1079 (M+Na); IR: 3521, 2883, 1195, 1053 cm^{-1}.

Compound **7**:

To a solution of **6** (3.92 g, 11.7 mmol) in 120 mL 1,4-dioxane was added pyridinium *p*-toluenesulfonate (150 mg, 0.60 mmol). The mixture was stirred for 24 hours at ambient temperature and then concentrated. Purification of the crude material by column chromatography (5% to 8% MeOH in CH_2Cl_2) afforded **7** (3.43 g, 9.71 mmol, 83%) as white crystals. m.p. = 61–63 °C (CH_2Cl_2); α_D (CH_3CN, c = 0.47): +49.7°; ^{1}H NMR (400 MHz, CD_3CN) δ: 5.22 (m, 1H), 5.02 (m, 1H), 4.89 (d, *J* = 2.3 Hz, 1H), 4.36–4.22 (m, 4H), 4.14 (d, *J* = 10.8 Hz, 1H), 4.07 (d, *J* = 10.8 Hz, 1H), 3.77 (d, *J* = 6.8 Hz, 1H), 3.43 (m, 4H), 2.92 (app s, 2H), 1.44 (d, *J* = 0.6 Hz, 3H), 1.31 (d, *J* = 0.6 Hz, 3H), 0.85 (s, 3H); ^{13}C NMR (101 MHz, CD_3CN) δ: 169.4, 143.5, 109.9, 100.4, 73.3, 71.8, 69.0, 66.0, 65.9, 65.1, 62.0, 41.6, 28.2, 22.1, 16.9; HRMS: m/z calcd. for $C_{15}H_{26}ClO_7^+$: 353.1362 (M+H); found: 353.1362 (M+H); IR: 3394, 2988, 1738, 1382, 1066 cm^{-1}

Compound **8**:

To a solution of **7** (2.40 g, 6.8 mmol) in 70 mL THF was added 2.0 M aqueous NaOH (20.5 mL, 41 mmol). The mixture was heated to 50 °C, stirred for a further 30 minutes, allowed to cool to room temperature, and concentrated. Purification of the crude material by column chromatography (66:33:2 v/v/v EtOAc:PhMe:AcOH) afforded **8** (1.05 g, 4.90 mmol, 72%) as white crystals. m.p. = 95–99 °C; α_D ($CHCl_3$, c = 1.69): +60.5°; ^{1}H NMR (400 MHz, $CDCl_3$) δ: 7.53 (br. s, 1H), 5.16 (m, 1H), 5.04 (m, 1H), 4.43 (d, *J* = 7.4 Hz, 1H), 4.39 (d, *J* = 14.0 Hz, 1H), 4.30 (d, *J* = 14.0 Hz, 1H), 3.62 (d, *J* = 4.5 Hz, 1H), 3.43 (dd, *J* = 7.4, 4.5 Hz, 1H), 1.37 (m, 6H); ^{13}C NMR (151 MHz, $CDCl_3$) δ: 172.8, 141.9, 109.0, 99.6, 68.8, 63.8, 57.4, 50.6, 27.3, 21.2;

HRMS: m/z calcd. for $C_{10}H_{14}O_5Na^+$: 237.0733 (M+Na); found: 237.0734 (M+Na); IR: 3650, 2375, 2993, 1723, 1378, 1200, 1152, 1064 cm^{-1}.

Compound **9**:

To a solution of **8** (1.05 g, 4.90 mmol) in 50 mL THF was added Et_3N (0.75 mL, 5.39 mmol). The mixture was cooled to –15 °C (ice / brine bath) and isopropyl chloroformate (2 M in toluene, 2.70 mL, 5.4 mmol) was added dropwise. The resulting cloudy suspension was stirred for 1 hour while naturally warming to 0 °C. The mixture was then diluted with 30 mL CH_3CN and 7.4 mL TMS-diazomethane (2 M in hexanes, 7.4 mL, 14.8 mmol) was added dropwise. The cooling bath was then removed, and the mixture was stirred for 24 hours at ambient temperature in the dark. The mixture was then treated with 0.6 mL glacial acetic acid, poured into 500 mL EtOAc, washed with NH_4Cl (250 mL), $NaHCO_3$ (250 mL), brine (250 mL), dried (Na_2SO_4), filtered, and concentrated. Purification of the crude material by column chromatography (30:70:1 to 50:50:1 v/v/v EtOAc:hexanes:Et_3N) afforded **9** (0.87 g, 3.65 mmol, 74%) a yellow/green oil. α_D (CH_3CN, c = 1.07): +41.4°; ^1H NMR (400 MHz, CD_3CN) δ: 5.77 (br. s, 1H), 5.09 (apparent q, *J* = 1.4 Hz, 1H), 5.03 (apparent q, *J* = 1.6 Hz, 1H), 4.37 (apparent dq, *J* = 14.0, 1.3 Hz, 1H), 4.25 (br. m, 1H), 4.23 (app. dq, *J* = 14.0, 1.3 Hz, 1H), 3.59 (br. d, *J* = 4.5 Hz, 1H), 3.31 (dd, *J* = 7.9, 4.5 Hz, 1H), 1.31 (d, *J* = 0.6 Hz, 3H), 1.29 (d, *J* = 0.6 Hz, 3H); ^{13}C NMR (101 MHz, CD_3CN) δ: 189.3, 144.3, 108.5, 100.0, 69.4, 65.2, 58.1, 56.4, 55.9, 27.5, 21.3; HRMS: m/z calcd. for $C_{11}H_{14}N_2O_4Na^+$: 261.0846 (M+Na); found: 261.0851 (M+Na); IR: 3085, 2990, 2110, 1637, 1372, 1074 cm^{-1}.

Compound **10**:

A solution of **9** (450 mg, 1.89 mmol) in 20 mL CH_2Cl_2 was treated with 4Å molecular sieves (ca. 30 beads) and stirred for 30 minutes. The mixture was then treated with $Rh_2(OAc)_4$ (17mg, 0.04 mmol) and stirred for six hours at ambient temperature. The mixture was then filtered through a pad of neutral alumina (CH_2Cl_2 rinse) and concentrated. The crude material was used directly in the next reaction. To obtain an analytical sample for characterization, a small portion of the crude material was purified by column chromatography (50:50:2 v/v/v pentane:EtOAc:Et_3N). α_D ($CHCl_3$, c = 0.54): –57.9°; ^1H NMR (400 MHz, $CDCl_3$) δ: 4.46 (dd, *J* = 3.3, 0.8 Hz, 1H), 3.90 (dd *J* = 11.6, 1.2 Hz, 1H), 3.50 (apparent t, *J* = 3.5 Hz, 1H), 3.25 (d, *J* = 11.6 Hz, 1H), 3.17 (dd, *J* = 3.6, 1.2 Hz, 1H), 1.60 (apparent ddt, *J* = 10.4, 5.4, 1.1 Hz, 1H), 1.53 (s, 3H), 1.48 (s, 3H), 1.18

(dd, J = 10.4, 5.7 Hz, 1H), 0.69 (apparent td, J = 5.5, 1.2 Hz, 1H); ^{13}C NMR (101 MHz, $CDCl_3$) δ: 199.1, 101.3, 64.6, 64.0, 53.3, 51.3, 25.9, 23.4, 23.0, 22.9, 16.8; HRMS: m/z calcd. for $C_{11}H_{15}O_4^+$: 211.0965 (M+H); found: 211.0970 (M+H); IR: 2993, 1703, 1383, 1223, 1088 cm^{-1}.

Compound **12**:

A cooled (0 ^{o}C) solution of crude epoxyketone **10** (360 mg) in 1 mL THF was treated with TBAF (1 M in THF, 8.0 mL, 8.0 mmol) and stirred for 8 hours at 0 °C. Purification of the crude material by adsorption onto silica gel followed by column chromatography (3:1 v/v EtOAc:pentane) afforded **12** (129 mg, 0.56 mmol, 30% over 2 steps based on 1.89 mmol diazoketone **9**) as a yellow oil. α_D ($CHCl_3$, c = 0.89) +34.0^o; ^{1}H NMR (400 MHz, $CDCl_3$) δ: 4.89 (dd, J = 48.4, 10.6 Hz, 1H), 4.77 (m, 1H), 4.33 (d, J = 12.4 Hz, 1H), 3.88 (m, 1H), 3.03 (d, J = 12.4 Hz, 1H), 2.65 (br. d, J = 9.4 Hz, 1H), 1.89 (m, 1H), 1.59 (s, 3H), 1.47 (s, 3H), 1.14 (dd, J = 6.4, 5.0 Hz, 1H) 1.07 (dd, J = 10.5, 6.4 Hz, 1H); ^{13}C NMR (101 MHz, $CDCl_3$) δ: 199.6 (d, J = 14.4 Hz), 100.4, 92.1 (d, J = 189.5 Hz), 69.4 (d, J = 9.1 Hz), 66.9 (d, J = 18.6 Hz), 65.4, 29.7 (d, J = 0.8 Hz), 29.5, 24.9 (d, J = 0.8 Hz), 19.0, 12.3; HRMS: m/z calcd. for $C_{11}H_{16}O_4F^+$: 231.1027 (M+H); found: 231.1038 (M+H); IR: 3442, 2925, 1714, 1382, 1228, 1198, 1098, 1075 cm^{-1}.

Compound **S3**:

A solution of **12** (114 mg, 0.49 mmol) in 5 mL CH_2Cl_2 was treated with pyridine (0.4 mL, 5.0 mmol), 4-dimethylaminopyridine (one crystal), and Ac_2O (0.19 mL, 2.0 mmol). The mixture was stirred for 4 hours at ambient temperature and was then diluted with 25 mL CH_2Cl_2, washed with NH_4Cl (10 mL), $NaHCO_3$ (10 mL), brine (10 mL), dried (Na_2SO_4), filtered, and concentrated. Purification of the crude material by column chromatography (1:1 / pentane:EtOAc) afforded **S3** (134 mg, 0.49 mmol, 99%) as a colourless oil. α_D ($CHCl_3$, c = 1.54): +100°; ^{1}H NMR (400 MHz, $CDCl_3$) δ: 5.11 (d, J = 1.4 Hz, 1H), 5.03 (apparent dd, J = 37.7, 11.0 Hz, 1H), 4.86 (m, 1H), 4.33 (d, J = 12.3 Hz, 1H), 2.99 (d, J = 12.3 Hz, 1H), 2.16 (s, 3H), 1.94 (m, 1H), 1.52 (d, J = 0.5 Hz, 1H), 1.45 (d, J = 0.5 Hz, 1H), 1.40 (dd, J = 6.6, 4.8 Hz, 1H), 1.13 (dd, J = 10.4, 6.6 Hz, 1H); ^{13}C NMR (151 MHz, $CDCl_3$) δ: 198.9 (d, J = 14.0 Hz), 170.4, 100.2, 88.3 (d, J = 190.7 Hz), 67.9 (d, J = 18.2 Hz), 67.7 (d, J = 8.8 Hz), 65.7, 29.9, 29.6, 26.2 (d, J = 0.8 Hz), 21.0 (d, J = 0.8 Hz), 18.9, 13.4; HRMS: m/z calcd. for $C_{13}H_{17}O_5FNa^+$: 295.0952 (M+Na); found: 295.0955; IR: 2923, 1740, 1721, 1376, 1233, 1089, 1071 cm^{-1}.

Compound **13**:

A cooled (–78 °C) solution of **S3** (134 mg, 0.49 mmol) in 5 mL THF was treated with L-selectride (1 M in THF, 0.49 mL, 0.49 mmol). The mixture was stirred at –78 °C for 15 minutes. Purification of the crude material by adsorption onto silica gel followed by column chromatography (3:2 v/v EtOAc:pentane) afforded **13** (113 mg, 0.41 mmol, 84%) as a white solid. m.p. = 162–166 °C; α_D ($CHCl_3$, c = 1.00): +115°; ^{1}H NMR (400 MHz, $CDCl_3$) δ: 5.07–4.90 (m, 2H), 4.69 (m, 2H), 4.17 (d, *J* = 12.3 Hz), 2.90 (d, *J* = 12.3 Hz), 2.20 (br. d, *J* = 3.8 Hz), 2.12 (s, 3H), 1.48 (s, 3H), 1.47 – 1.40 (m, 1H), 1.44 (s, 3H), 1.11 (dd, *J* = 6.2, 5.2 Hz), 0.48 (ddd, *J* = 9.4, 6.2, 1.3 Hz, 1H); ^{13}C NMR (151 MHz, $CDCl_3$) δ: 170.7, 99.2, 88.0 (d, *J* = 180.2 Hz), 69.0 (d, *J* = 7.0 Hz), 66.8, 66.4 (d, *J* = 17.1 Hz), 64.3 (d, *J* = 17.2 Hz), 29.6, 23.3, 22.6 (d, *J* = 5.8 Hz), 21.2, 19.1, 7.8; HRMS: m/z calcd. for $C_{13}H_{20}O_5F^+$: 275.1289 (M+H); found: 273.1311 (M+H); IR: 3475, 2996, 1740, 1376, 1240, 1107 cm^{-1}.

Compound **14**:

A solution of **13** (110 mg, 0.40 mmol) and quinuclidine (445 mg, 4.0 mmol) in 1.0 mL DMF was treated with 4Å molecular sieves (ca. 10 beads) and stirred for 30 minutes. The mixture was then treated with a solution of 2,4-dinitrofluorobenzene (298 mg, 1.6 mmol) in 0.5 mL DMF. The resulting dark-green solution was stirred for 24 hours at ambient temperature and then diluted with 20 mL EtOAc, washed with NH_4Cl (10 mL), $NaHCO_3$ (10 mL), brine (10 mL), dried (Na_2SO_4), filtered, and concentrated. Purification of the crude material by adsorption onto silica gel followed by column chromatography (3:2 to 1:1 v/v pentane:EtOAc) afforded **14** (143 mg, 0.33 mmol, 81%) as a white solid. m.p. = 149–153 °C; α_D ($CHCl_3$, c = 2.15): +204°; ^{1}H NMR (400 MHz, $CDCl_3$) δ: 8.77 (d, *J* = 2.8 Hz, 1H), 8.41 (dd, *J* = 9.4, 2.8 Hz, 1H), 7.27 (d, *J* = 9.4 Hz, 1H), 5.48 (ddd, *J* = 7.7, 4.8, 2.8 Hz, 1H), 5.29 (ddd, *J* = 47.5, 11.0, 4.8, 4.8 Hz, 1H), 5.16 (ddd, *J* = 11.0, 5.0, 3.2 Hz, 1H), 4.85 (apparent t, *J* = 3.1 Hz, 1H), 4.22 (d, *J* = 12.5 Hz, 1H), 2.88 (d, *J* = 12.5 Hz, 1H), 2.12 (s, 3H), 1.59 (m, 1H), 1.50 (s, 3H), 1.46 (s, 3H), 1.21 (dd, *J* = 6.5, 5.0 Hz, 1H), 0.55 (ddd, *J* = 9.3, 6.5, 1.1 Hz, 1H); ^{13}C NMR (151 MHz, $CDCl_3$) δ: 170.2, 155.9, 140.6, 139.5, 129.0, 122.3, 116.1 (d, *J* = 2.3 Hz), 99.4, 85.7 (d, *J* = 191.8 Hz), 73.9 (d, *J* = 15.6 Hz), 68.2 (d, *J* = 6.8 Hz), 66.4, 65.9 (d, *J* = 16.8 Hz), 29.5, 24.4, 21.5 (d, *J* = 5.6 Hz), 21.0, 18.9, 9.0; HRMS: m/z calcd. for $C_{19}H_{22}O_9N_2F^+$: 441.1304 (M+H); found: 441.1291 (M+H); IR: 2923, 1744, 1607, 1534, 1345, 1275, 1239 cm^{-1}.

Compound **S4**:

A cooled (0 ^{o}C) solution of **14** (136 mg, 0.31 mmol) in 6.2 mL MeOH was treated with K_2CO_3 (64 mg, 0.46 mmol) and stirred at 0 °C for 30 minutes. The resulting orange solution was diluted with CH_2Cl_2 (20 mL), washed with NH_4Cl (10 mL), $NaHCO_3$ (10 mL), brine (10 mL), dried (Na_2SO_4), filtered, and concentrated. Purification of the crude material by column chromatography (1:1 / pentane:EtOAc) afforded **S4** (113 mg, 0.28 mmol, 92%) as a white solid. m.p. = 176–180 °C; α_D ($CHCl_3$, c = 0.63): +179°; ^{1}H NMR (600 MHz, $CDCl_3$) δ: 8.76 (d, *J* = 2.7 Hz, 1H), 8.42 (dd, *J* = 9.2, 2.7 Hz, 1H), 7.27 (d, *J* = 9.2 Hz, 1H), 5.43 (m, 1H), 5.01 (ddd, *J* = 48.0, 10.5, 4.6 Hz, 1H), 4.71 (apparent t, *J* = 3.3 Hz, 1H), 4.21 (d, *J* = 12.5 Hz, 1H), 4.02 (m, 1H), 2.94 (d, *J* = 12.5 Hz, 1H), 2.52 (br. d, *J* = 9.1 Hz, 1H), 1.59 – 1.54 (m, 1H), 1.57 (s, 3H), 1.48 (s, 3H), 1.06 (dd, *J* = 6.3, 5.3 Hz, 1H), 0.52 (ddd, J = 9.4, 6.3, 0.9 Hz, 1H); ^{13}C NMR (151 MHz, $CDCl_3$) δ: 155.8, 140.5, 139.5, 129.0, 122.3, 115.8 (d, *J* = 1.7 Hz), 99.7, 88.6 (d, *J* = 188.2 Hz), 73.9 (d, *J* = 16.0 Hz), 69.9 (d, *J* = 7.1 Hz), 66.2, 64.2 (d, *J* = 18.3 Hz), 29.4, 23.9, 22.1 (d, *J* = 5.9 Hz), 19.1, 8.8; HRMS: m/z calcd. for $C_{17}H_{19}N_2O_8FNa^+$: 421.1018 (M+Na); found: 421.1018; IR: 3545, 2995, 1607, 1531, 1345, 1280, 1098 cm^{-1}.

(1*R*,2*S*,3*S*,4*R*,5*S*,6*S*)-5-(2,4-dinitrophenoxy)-4-fluoro-1-(hydroxymethyl)bicyclo[4.1.0]heptane-2,3-diol (3):

A solution of **S4** (110 mg, 0.28 mmol) in 6 mL methanol was treated with Amberlite™ IR 120 H^+ resin (ca. 100 beads) and stirred at ambient temperature for 24 hours. The mixture was then filtered and concentrated. Purification of the crude material by adsorption onto Celite® followed by reversed phase column chromatography (0 to 100% CH_3CN in H_2O) afforded **3** (80.2 mg, 0.22 mmol, 81%) as a white solid. m.p. = 186–190 °C; α_D ($CHCl_3$, c = 1.54): +211°; ^{1}H NMR (400 MHz, CD_3CN) δ: 8.67 (d, *J* = 2.8 Hz, 1H), 8.43 (dd, *J* = 9.4, 2.8 Hz, 1H), 7.49 (d, *J* = 9.4 Hz, 1H), 5.51 (m, 1H), 4.80 (ddd, *J* = 47.7, 10.5, 4.8 Hz, 1H), 4.40 (m, 1H), 3.82 (m, 1H), 3.79 (d, *J* = 3.1 Hz, 1H), 3.51 (dd, *J* = 11.4, 7.4 Hz, 1H), 3.50 (d, *J* = 6.3 Hz, 1H), 3.43 (dd, *J* = 11.4, 4.5 Hz, 1H), 3.02 (dd, *J* = 7.4, 4.5 Hz, 1H), 1.62 (m, 1H), 0.71 (apparent td, J = 5.4, 0.7 Hz, 1H), 0.61 (ddd, *J* = 9.4, 5.5, 1.4 Hz, 1H); ^{13}C NMR (151 MHz, CD_3CN) δ: 156.3, 141.3, 140.4, 129.9, 122.4, 117.6, 89.4 (d, *J* = 186.0 Hz), 74.9 (d, *J* = 15.5 Hz), 71.9 (d, *J* = 7.2 Hz), 66.9, 65.7 (d, *J* = 17.2 Hz), 30.4 (d, *J* = 1.1 Hz), 19.4 (d, *J* = 5.6 Hz), 11.0; HRMS: m/z calcd. for $C_{14}H_{15}N_2O_8FNa^+$: 381.0705 (M+Na); found: 381.0702; IR: 3391, 2928, 1607, 1530, 1347, 1284, 1082 cm^{-1}.

Protein expression and purification

A plasmid containing the gene encoding *Tm*GalA in a pET28a vector, which has been described previously[1], was transformed into *E. coli* BL21 (DE3) cells. Cultures were grown at 37 °C in Luria-Bertani broth containing kanamycin (50 μg/mL), until an optical density at 600 nm of approximately 0.6 absorbance units was reached. Over-expression was induced by the addition of 0.5 mM isopropyl β-D-1-thiogalactopyranoside (IPTG), and cells were cultured for a further 4 h at 37 °C. Cells were harvested by centrifugation, re-suspended in phosphate buffered saline (PBS), pH 7.4, 20 mM imidazole, and lysed using a cell disruptor at 30 kpsi. *Tm*GalA was applied to a nickel affinity chromatography column (5 mL HisTrap FF, GE Healthcare), washed with 10 column volumes of PBS, pH 7.4, 50 mM imidazole and eluted with 5 column volumes of PBS, pH 7.4, 250 mM imidazole. *Tm*GalA was buffer exchanged into 20 mM HEPES, pH 7.4, 150 mM NaCl (HiPrep 26/10 desalting column, GE Healthcare), and then applied to a size exclusion column (Superdex 200 16/60, GE Healthcare) for further purification. *Tm*GalA was judged to be >95% pure by SDS-PAGE.

Crystallisation

*Tm*GalA (10 mg/ml) was crystallised from 0.2 M $MgSO_4$ and 20% (w/v) poly(ethylene glycol) (PEG) 3350. Crystals were soaked in 1 mM **1**, 30% (w/v) PEG 3350 for between 1 h and 7 days, which also acted as the cryo-protectant prior to freezing in liquid nitrogen. Prior to crystallisation with **3**, *Tm*GalA (2 mg/ml) in 50 mM HEPES, pH 7.4 was incubated with 0.45 mM **3** at 60 °C overnight and then buffer exchanged into 10 mM HEPES, pH 7.4, 150 mM NaCl to remove unbound inhibitor and the dinitrophenol product using a PD10 desalting column (GE Healthcare). *Tm*GalA (8.7 mg/ml) was crystallised from 2.0 M ammonium sulphate and 0.13 M ammonium citrate. The crystals were cryoprotected in 2.25 M ammonium sulfate, 20% (w/v) glycerol and frozen in liquid nitrogen.

Data collection and processing

X-ray diffraction data were collected at Diamond Light Source on beamlines I24 for *Tm*GalA native crystals, I04-1 for *Tm*GalA in complex with **1** and **2**, and I02 for *Tm*GalA in complex with **3**. Diffraction data were processed using the Xia2[2] pipeline to run distl[3], labelit[4], XDS[5], and Pointless[6] programs from the CCP4 suite[7]. Molecular replacement was performed using MOLREP[8] with Protein Data Bank (PDB) entry 1ZY9 as the search model for the *Tm*GalA

native structure, and subsequently this structure was used as the search model for *Tm*GalA in complex with **1**, **2**, and **3**. Refinement was performed using REFMAC5[9] and manual model building was done using Coot[10]. Structures were optimised using PDB_REDO[11]. Models for the inhibitors were built in Chem3D Pro 14.0 (Cambridgesoft, UK), and the library generated with PRODRG[12].

Enzyme inactivation kinetics

The kinetic parameters K_i and k_{inact} for inhibition of *T. maritima* α-galactosidase, were determined using a classical dilution assay method that entailed incubating the enzyme with a range of concentration of inactivator (**3**) at 60 °C in 50 mM HEPES buffer, pH 7.4. At specified time points aliquots were taken from the inactivation stock solution and upon incubation in a cuvette at 25 °C for 5 min they were diluted with 100 μM 4-nitrophenyl α-D-galactopyranoside in 50 mM HEPES buffer, pH 7.4. Enzyme activity was monitored at 400 nm using the Cary 6000i UV-Visible-NIR spectrophotometer. Pseudo first-order rate constants for inactivation (k_{obs}) at each inactivator concentration were determined by fitting the absorbance versus time data to a standard first-order rate equation using a non-linear least squares routine in a computer program (Prism 4.0). The first- and second-order rate constants (k_{inact} and k_{inact}/K_i, respectively) for the inactivation process were determined by fitting the k_{obs} versus inactivator concentration data to a standard Michaelis–Menten expression.

Enzyme reactivation kinetics

In a typical enzyme reactivation assay, *Tm*GalA was incubated with the inactivator (**3**) at 60 °C in 50 mM HEPES buffer, pH 7.4. After incubating the sample for 5 hours at 60 °C, excess inactivator was removed using a 30K molecular weight cut-off centrifugal filter. Following 5 successive washes at 4 °C with 50 mM HEPES buffer, pH 7.4, the sample volume was made up to a volume of 300μL and incubated at 60 °C. At specified time points aliquots were taken from the reactivation stock solution and upon incubation in a cuvette at 25 °C for 5 min they were diluted with 100 μM 4-nitrophenyl α-D-galactopyranoside in 50 mM HEPES buffer, pH 7.4 and enzyme activity was monitored at 400 nm. No reactivation was apparent after 5 days of incubation.

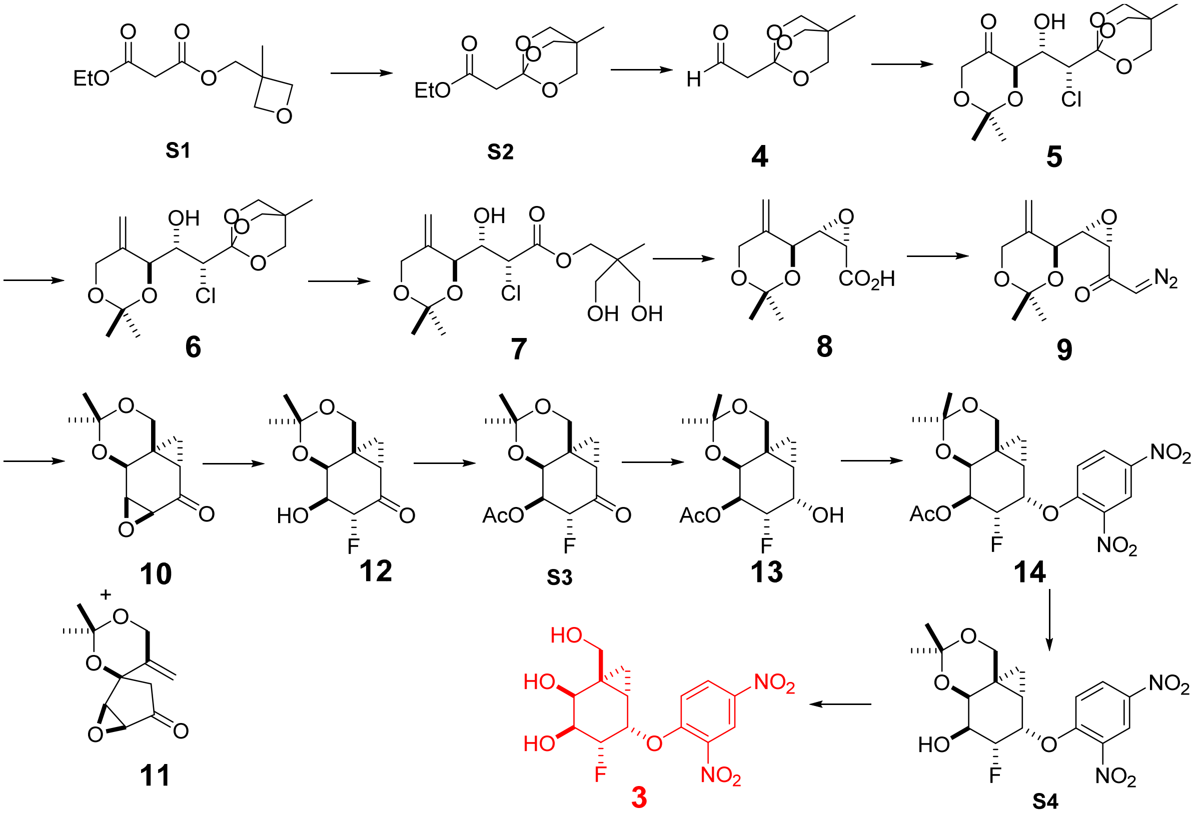

Scheme S1: Synthetic route used to make mechanism-based in activator **3**. Compounds that have structures shown in the main text are denoted by large font numerals, while all other structures are labelled with a smaller font.

Table S1. Data processing and refinement statistics.

	Native *Tm*GalA	*Tm*GalA in complex with **1**	*Tm*GalA in complex with **2**	*Tm*GalA in complex with **3**
Beamline[a]	I24	I04–1	I04–1	I02
Wavelength	0.97	0.92	0.92	0.98
Resolution (Å)[b]	32.09–1.80 (1.85–1.80)	43.69–1.53 (1.57–1.53)	55.66–1.62 (1.66–1.62)	48.79–1.55 (1.58–1.55)
Completeness[b]	99.0 (99.2)	98.4 (99.1)	99.6 (99.7)	100 (99.9)
Multiplicity[b]	6.4 (6.4)	4.9 (4.9)	5.1 (4.6)	6.5 (6.6)
Mean I/σI[b]	14.9 (2.6)	23.1 (2.5)	15.8 (2.4)	12.6 (2.0)
R_{merge}[b]	0.079 (0.706)	0.035 (0.675)	0.055 (0.594)	0.068 (0.864)
R_{pim}[b]	0.037 (0.328)	0.022 (0.399)	0.031 (0.339)	0.042 (0.542)
Wilson B factor	21.4	18.7	18.0	17.4
Total observations	379823	474234	416664	596003
Total unique observations	59066	95993	82206	92049
Space group	$P2_12_12_1$	$P2_12_12_1$	$P2_12_12_1$	$P2_12_12_1$
Unit cell parameters *a*, *b*, *c*	68.0, 95.8, 97.5	68.2, 96.0, 98.1	68.3, 96.0, 98.0	66.9, 96.2, 97.6
Unit cell parameters α, β, γ	90.0, 90.0, 90.0	90.0, 90.0, 90.0	90.0, 90.0, 90.0	90.0, 90.0, 90.0
R_{cryst} (%)	18.6	19.3	17.2	15.4
R_{free} (%)	23.1	22.2	19.8	18.2
RMSD bonds (Å)[c]	0.017	0.015	0.015	0.015
RMSD angles (°)[c]	1.79	1.57	1.61	1.68
PDB code	5M0X	5M12	5M16	5M1I

[a] Beamlines at Diamond Light Source; [b] Values in parentheses represent outer shell; [c] RMSD, root mean square deviation

References

[1] S. Chakladar, Y. Wang, T. Clark, L. Cheng, S. Ko, D. J. Vocadlo, A. J. Bennet, *Nat Commun* **2014**, *5*, 5590.

[2] G. Winter, *J Appl Crystallogr* **2009**, *43*, 186-190.

[3] Z. Zhang, N. K. Sauter, H. van den Bedem, G. Snell, A. M. Deacon, *J Appl Crystallogr* **2006**, *39*, 112-119.

[4] N. K. Sauter, R. W. Grosse-Kunstleve, P. D. Adams, *J Appl Crystallogr* **2004**, *37*, 399-409.

[5] W. Kabsch, *Acta Crystallogr D Biol Crystallogr* **2010**, *66*, 125-132.

[6] P. Evans, *Acta Crystallogr D Biol Crystallogr* **2006**, *62*, 72-82.

[7] Collaborative Computational Project Number 4, *Acta Crystallogr D Biol Crystallogr* **1994**, *50*, 760-763.

[8] A. Vagin, A. Teplyakov, *J Appl Crystallogr* **1997**, *30*, 1022-1025.

[9] G. N. Murshudov, P. Skubak, A. A. Lebedev, N. S. Pannu, R. A. Steiner, R. A. Nicholls, M. D. Winn, F. Long, A. A. Vagin, *Acta Crystallogr D Biol Crystallogr* **2011**, *67*, 355-367.

[10] P. Emsley, K. Cowtan, *Acta Crystallogr D Biol Crystallogr* **2004**, *60*, 2126-2132.

[11] R. P. Joosten, F. Long, G. N. Murshudov, A. Perrakis, *IUCrJ* **2014**, *1*, 213-220.

[12] A. W. Schuttelkopf, D. M. van Aalten, *Acta Crystallogr D Biol Crystallogr* **2004**, *60*, 1355-1363.

NMR spectra for all new compounds

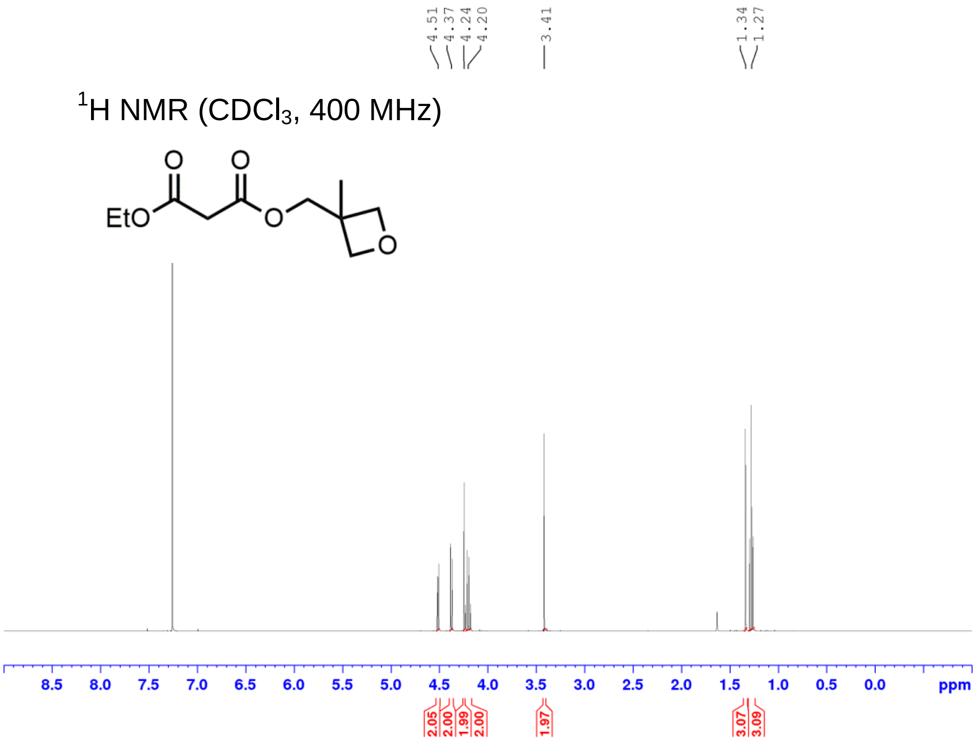

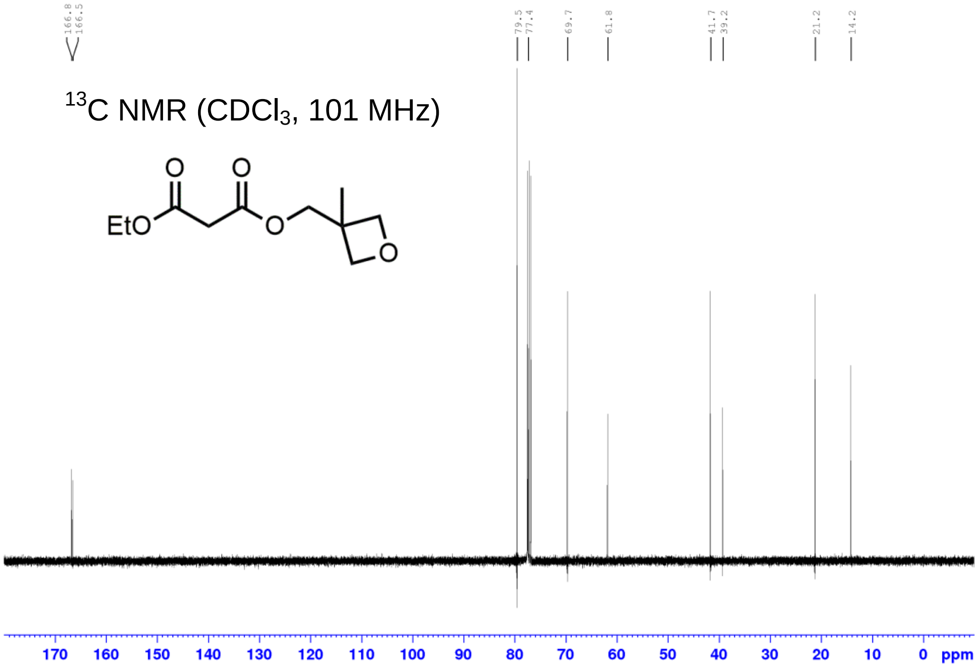

^{1}H NMR (CD_3CN, 400 MHz)

ethyl 2-(4-methyl-2,6,7-trioxabicyclo[2.2.2]octan-1-yl)acetate

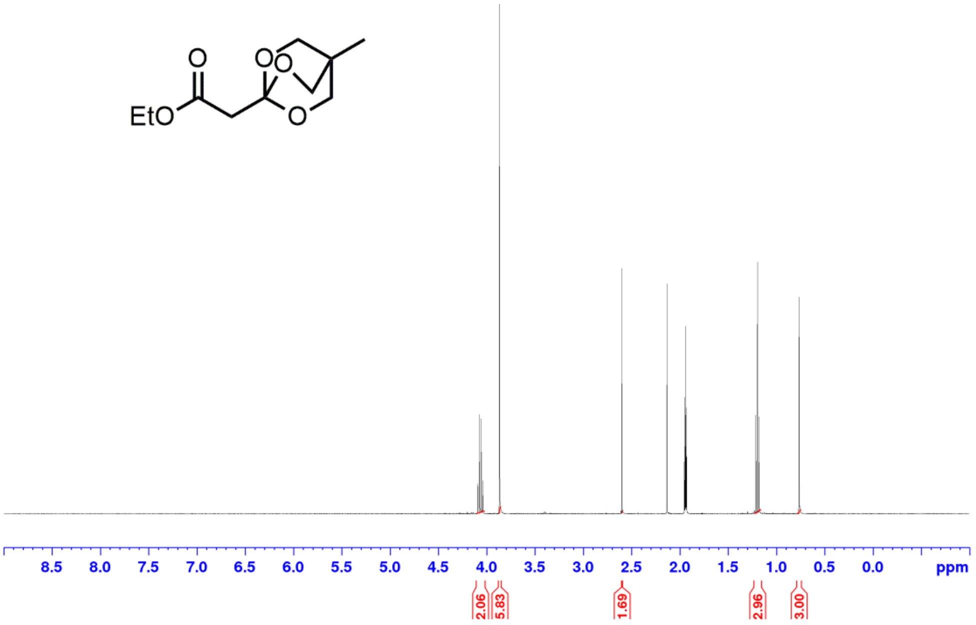

^{13}C NMR (CD_3CN, 126 MHz)

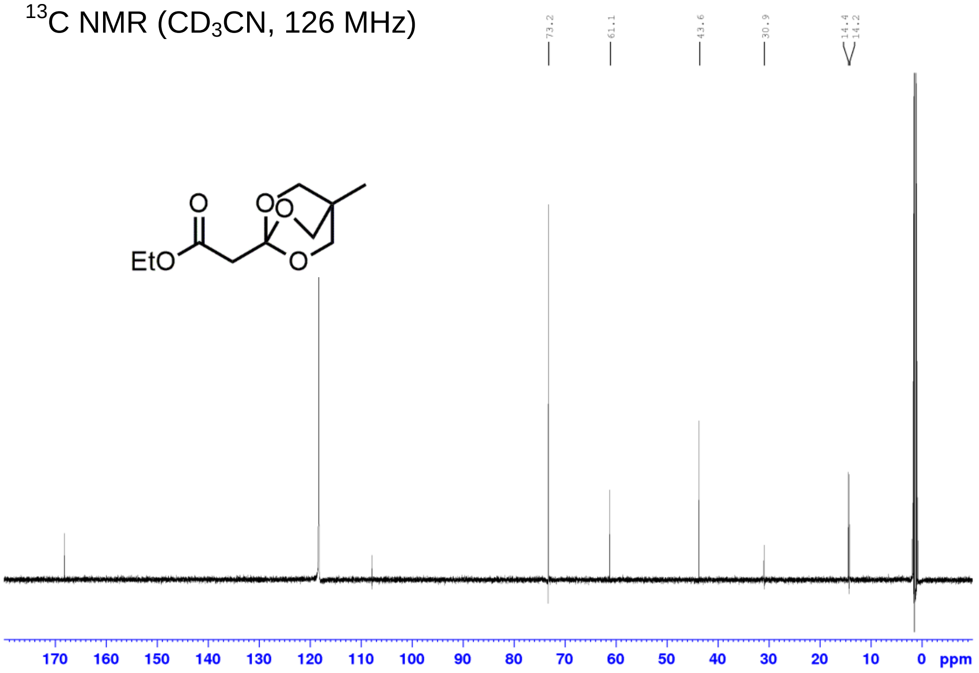

^{1}H NMR (CD_3CN, 400 MHz)

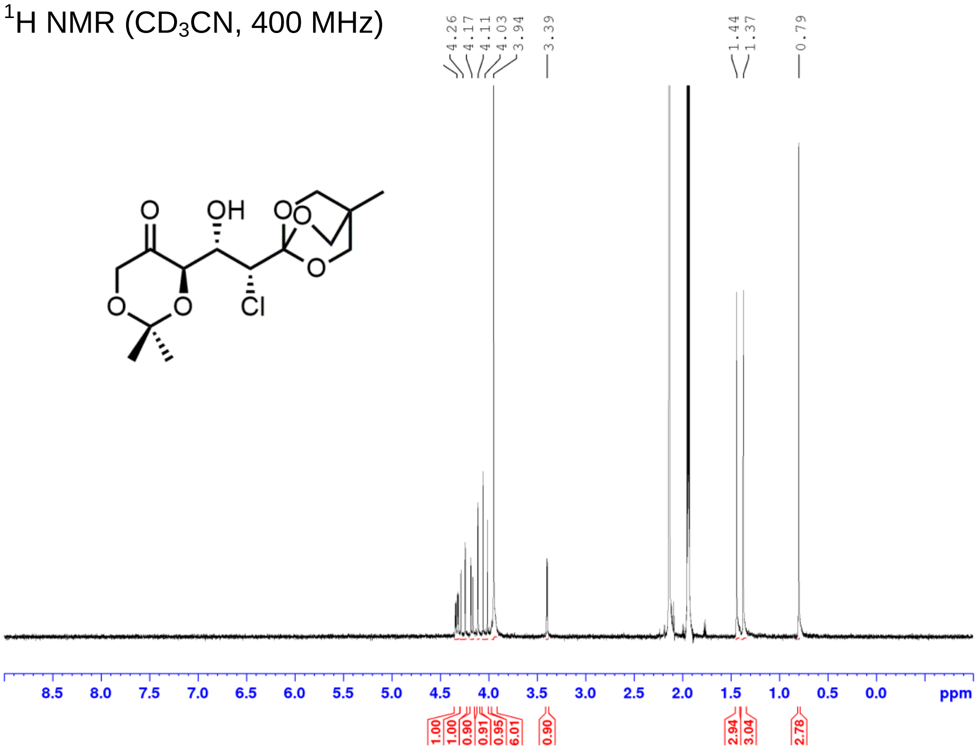

^{13}C NMR (CD_3CN, 101 MHz)

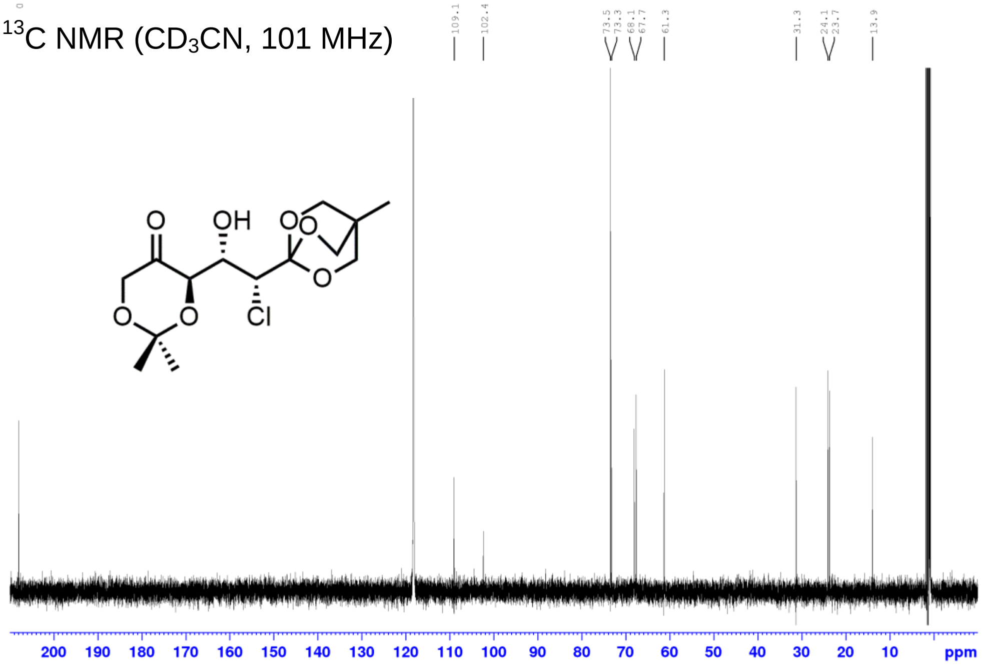

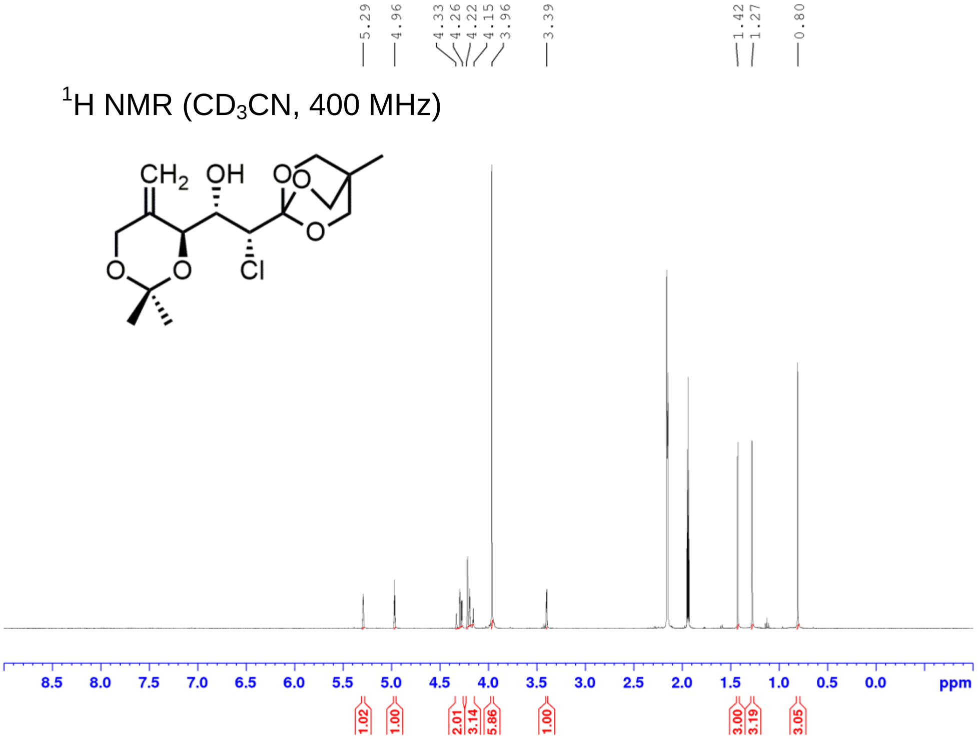

5.29
4.96
4.33
4.26
4.22
4.15
3.96
3.39
1.42
1.27
0.80
1H NMR (CD3CN, 400 MHz)
CH2
OH
O
O
O
O
O
Cl
8.5 8.0 7.5 7.0 6.5 6.0 5.5 5.0 4.5 4.0 3.5 3.0 2.5 2.0 1.5 1.0 0.5 0.0 ppm
1.02
1.00
2.01
3.14
5.86
1.00
3.00
3.19
3.05

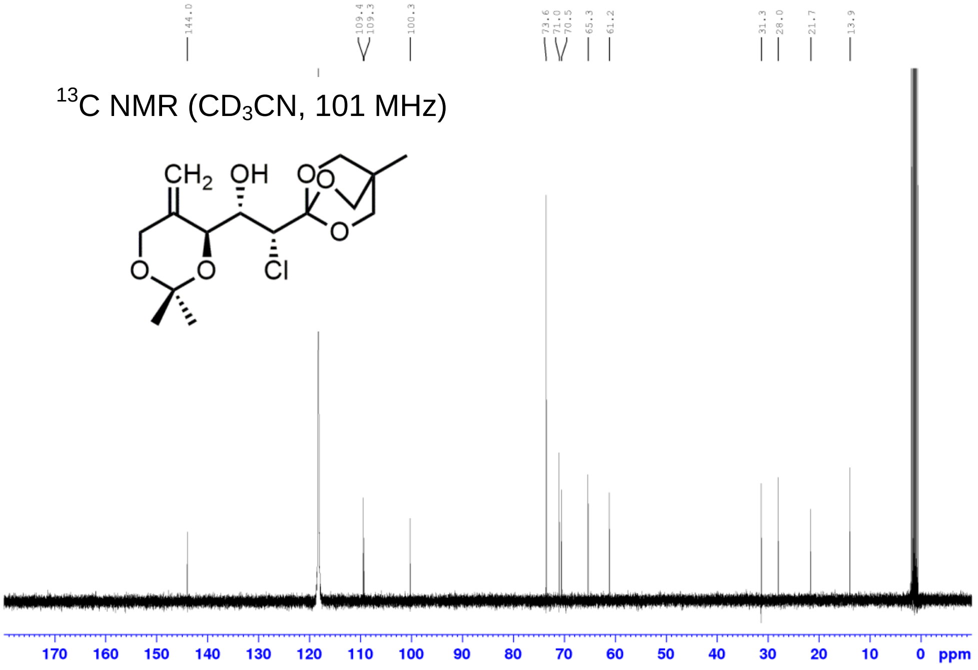

144.0
109.4
109.3
100.3
73.6
71.0
70.5
65.3
61.2
31.3
28.0
21.7
13.9
13C NMR (CD3CN, 101 MHz)
CH2
OH
O
O
O
O
O
Cl
170 160 150 140 130 120 110 100 90 80 70 60 50 40 30 20 10 0 ppm

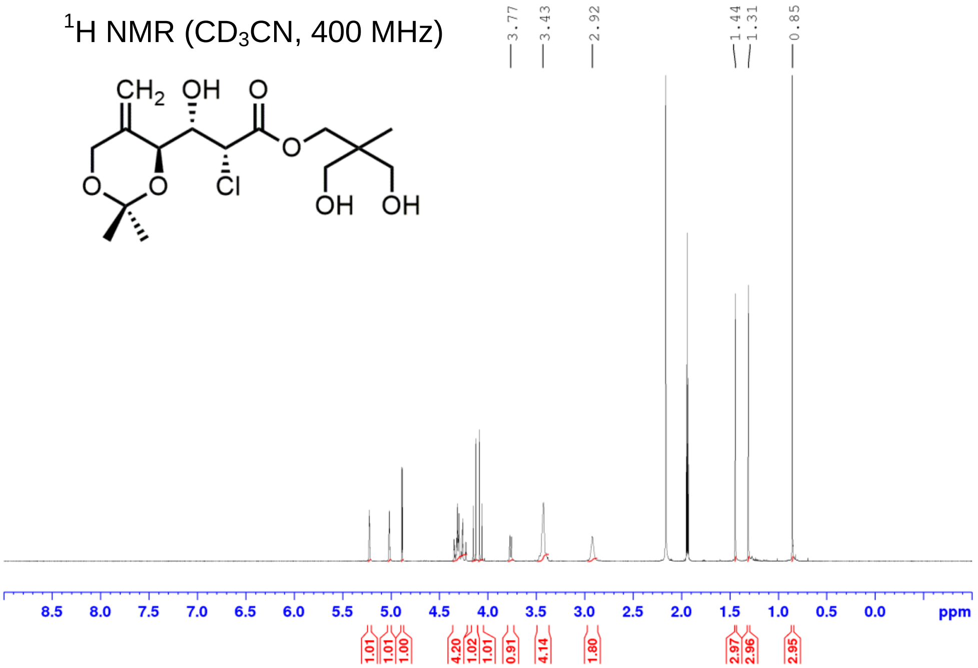

^{1}H NMR (CD_3CN, 400 MHz)
CH2 OH O
O O Cl OH OH
3.77
3.43
2.92
1.44
1.31
0.85
8.5 8.0 7.5 7.0 6.5 6.0 5.5 5.0 4.5 4.0 3.5 3.0 2.5 2.0 1.5 1.0 0.5 0.0 ppm
1.01 1.01 1.00 4.20 1.02 1.01 0.91 4.14 1.80 2.97 2.96 2.95

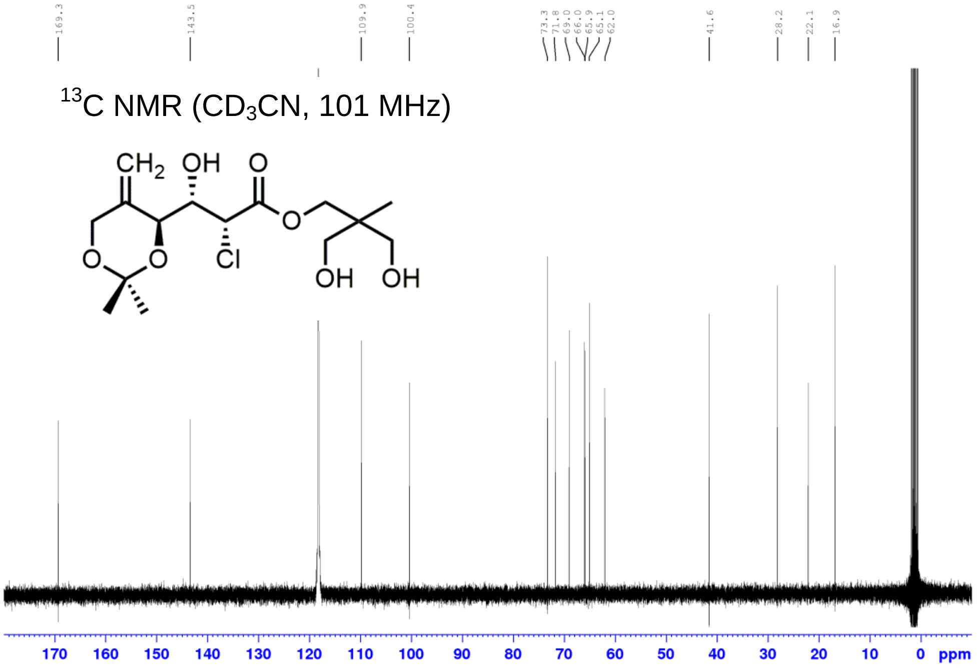

169.3
143.5
109.9
100.4
73.3
71.8
69.0
66.0
65.9
65.1
62.0
41.6
28.2
22.1
16.9
^{13}C NMR (CD_3CN, 101 MHz)
CH2 OH O
O O Cl OH OH
170 160 150 140 130 120 110 100 90 80 70 60 50 40 30 20 10 0 ppm

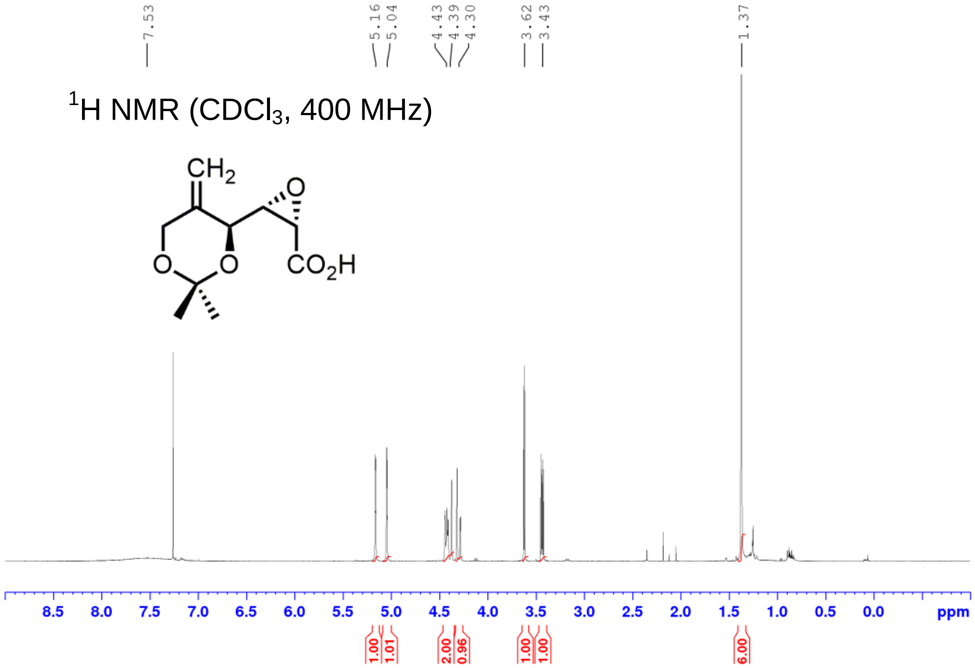
7.53
5.16
5.04
4.43
4.39
4.30
3.62
3.43
1.37
^{1}H NMR ($CDCl_3$, 400 MHz)
CH2
O
O
O
CO2H
8.5
8.0
7.5
7.0
6.5
6.0
5.5
5.0
4.5
4.0
3.5
3.0
2.5
2.0
1.5
1.0
0.5
0.0
ppm
1.00
1.01
2.00
0.96
1.00
1.00
6.00

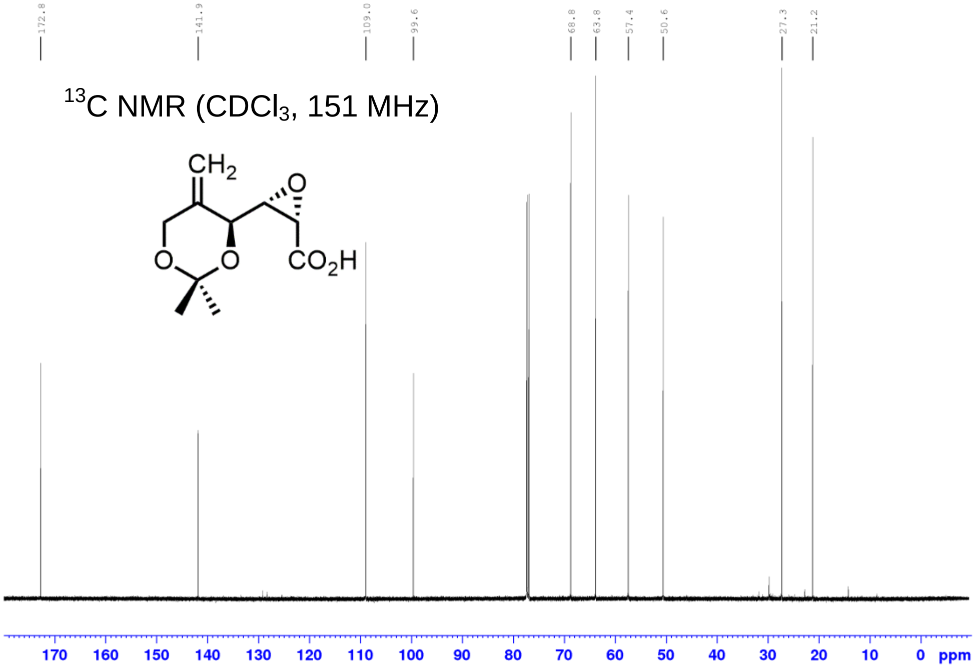
172.8
141.9
109.0
99.6
68.8
63.8
57.4
50.6
27.3
21.2
^{13}C NMR ($CDCl_3$, 151 MHz)
CH2
O
O
O
CO2H
170
160
150
140
130
120
110
100
90
80
70
60
50
40
30
20
10
0
ppm

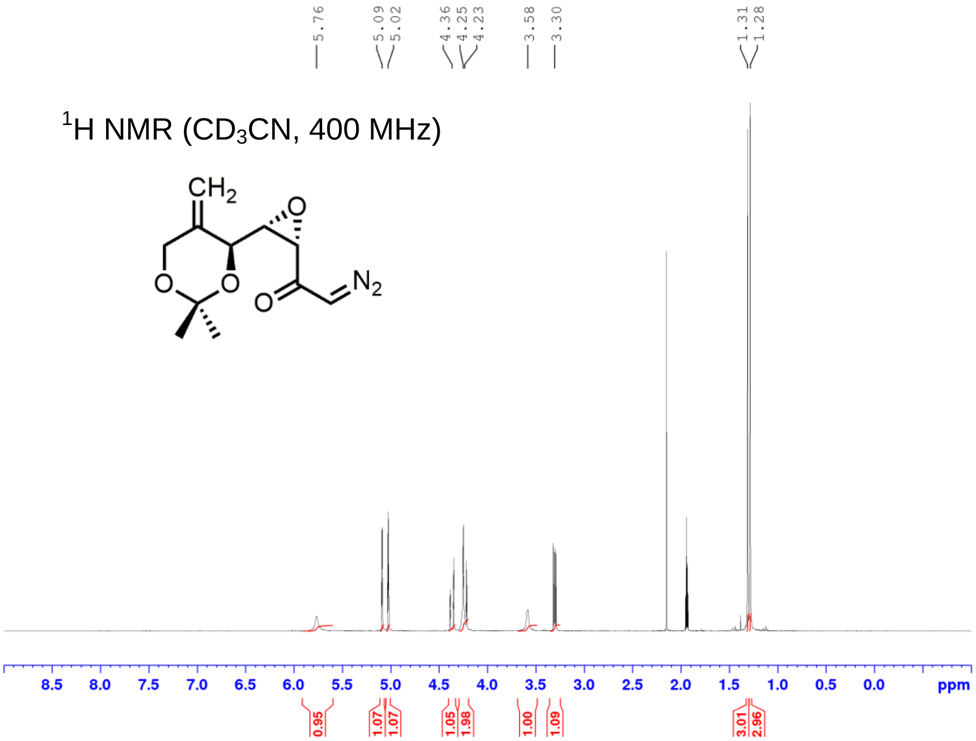
5.76
5.09
5.02
4.36
4.25
4.23
3.58
3.30
1.31
1.28
^{1}H NMR (CD_3CN, 400 MHz)
CH_2
O
O
O
O
N_2
8.5 8.0 7.5 7.0 6.5 6.0 5.5 5.0 4.5 4.0 3.5 3.0 2.5 2.0 1.5 1.0 0.5 0.0 ppm
0.95
1.07
1.07
1.05
1.98
1.00
1.09
3.01
2.96

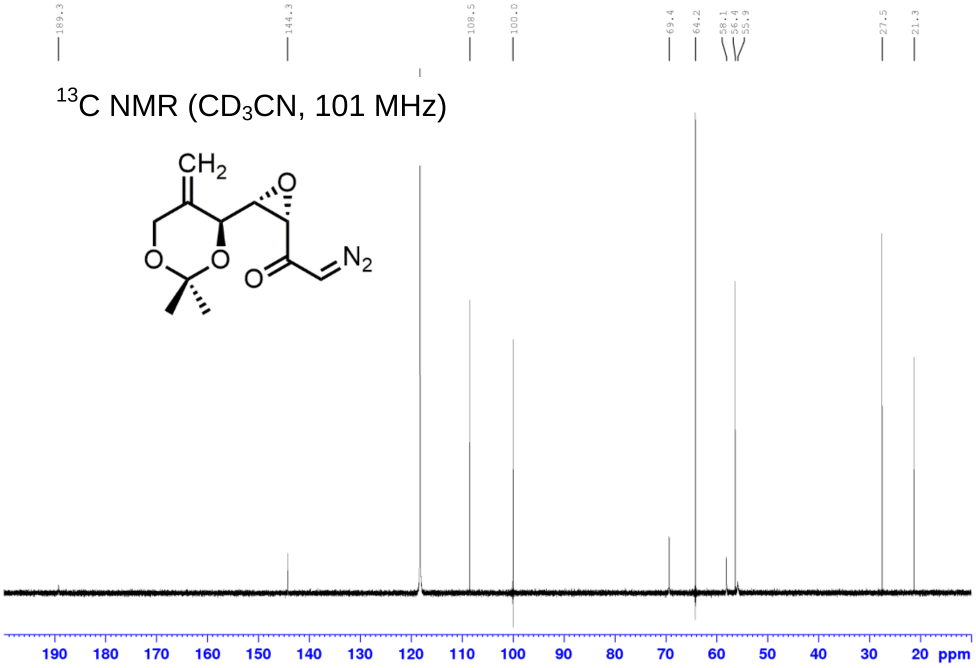
189.3
144.3
108.5
100.0
69.4
64.2
58.1
56.4
55.9
27.5
21.3
^{13}C NMR (CD_3CN, 101 MHz)
CH_2
O
O
O
O
N_2
190 180 170 160 150 140 130 120 110 100 90 80 70 60 50 40 30 20 ppm

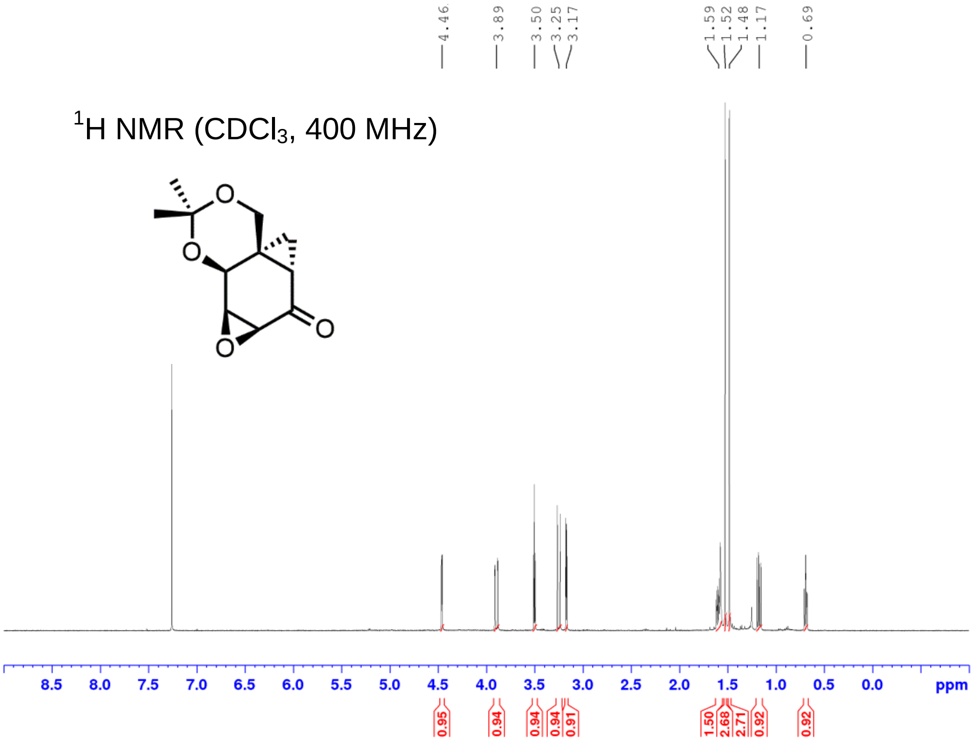

4.46
3.89
3.50
3.25
3.17
1.59
1.52
1.48
1.17
0.69
^{1}H NMR ($CDCl_3$, 400 MHz)
8.5 8.0 7.5 7.0 6.5 6.0 5.5 5.0 4.5 4.0 3.5 3.0 2.5 2.0 1.5 1.0 0.5 0.0 ppm
0.95 0.94 0.94 0.94 0.91 1.50 2.68 2.71 0.92 0.92

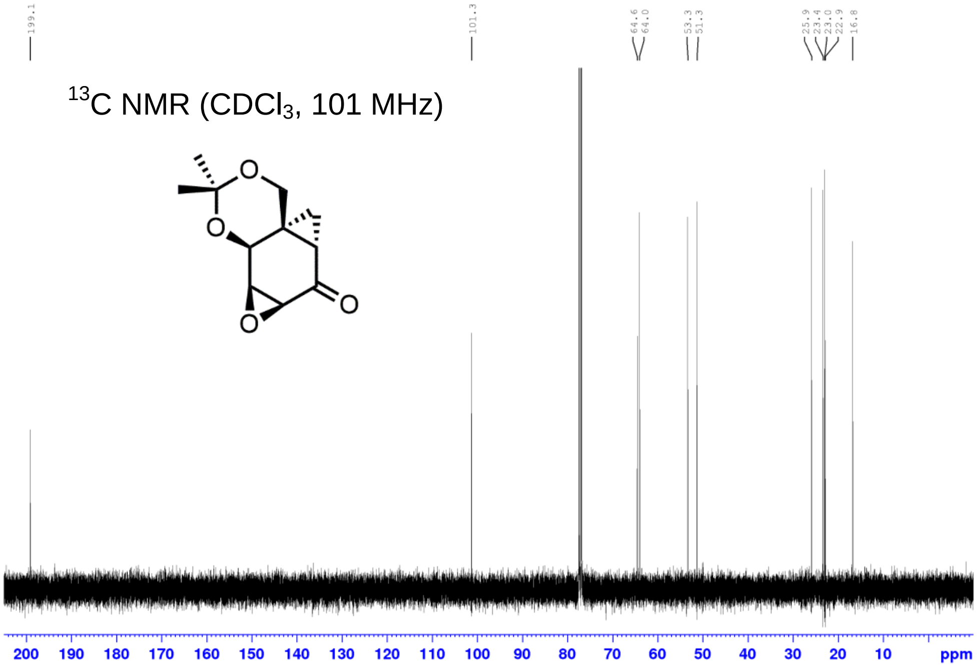

199.1
101.3
64.6
64.0
53.3
51.3
25.9
23.4
23.0
22.9
16.8
^{13}C NMR ($CDCl_3$, 101 MHz)
200 190 180 170 160 150 140 130 120 110 100 90 80 70 60 50 40 30 20 10 ppm

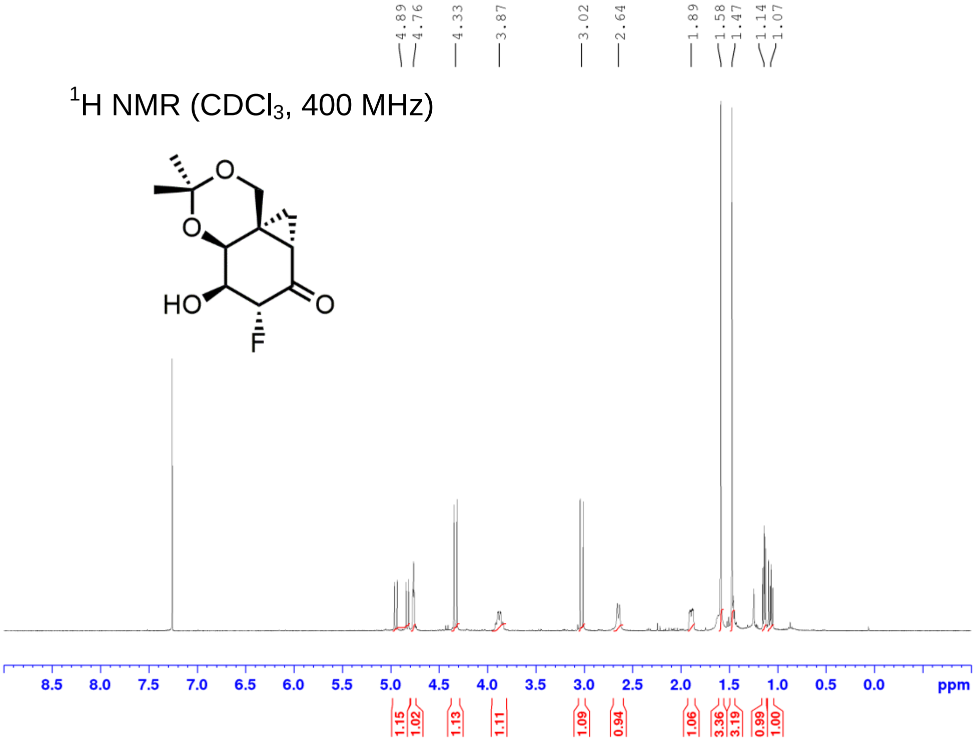
^{1}H NMR ($CDCl_3$, 400 MHz)
HO
F
O
4.89 4.76 4.33 3.87 3.02 2.64 1.89 1.58 1.47 1.14 1.07
8.5 8.0 7.5 7.0 6.5 6.0 5.5 5.0 4.5 4.0 3.5 3.0 2.5 2.0 1.5 1.0 0.5 0.0 ppm
1.15 1.02 1.13 1.11 1.09 0.94 1.06 3.36 3.19 0.99 1.00

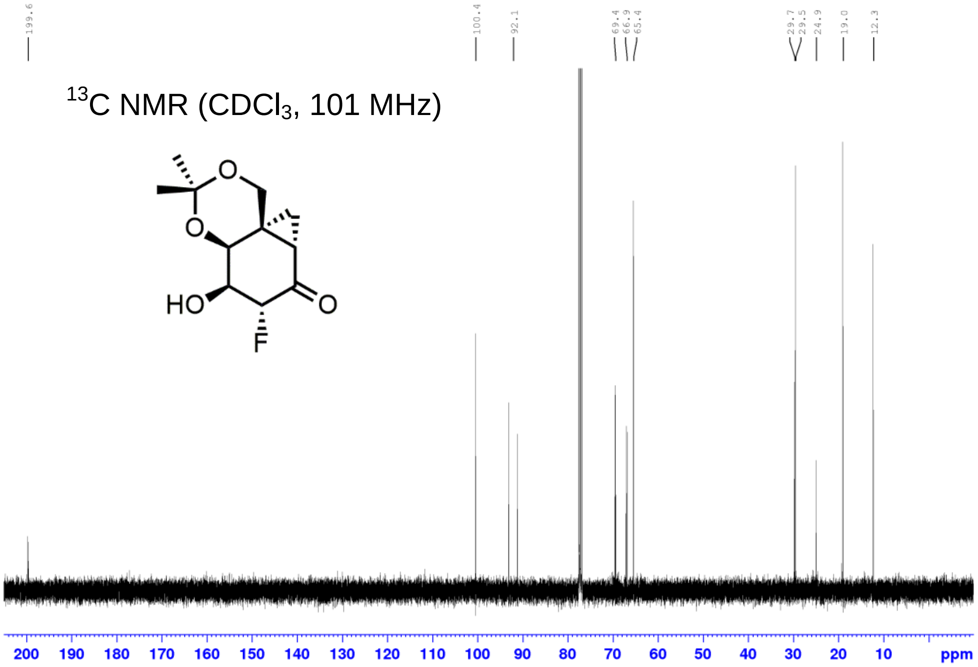
^{13}C NMR ($CDCl_3$, 101 MHz)
HO
F
O
199.6 100.4 92.1 69.4 66.9 65.4 29.7 29.5 24.9 19.0 12.3
200 190 180 170 160 150 140 130 120 110 100 90 80 70 60 50 40 30 20 10 ppm

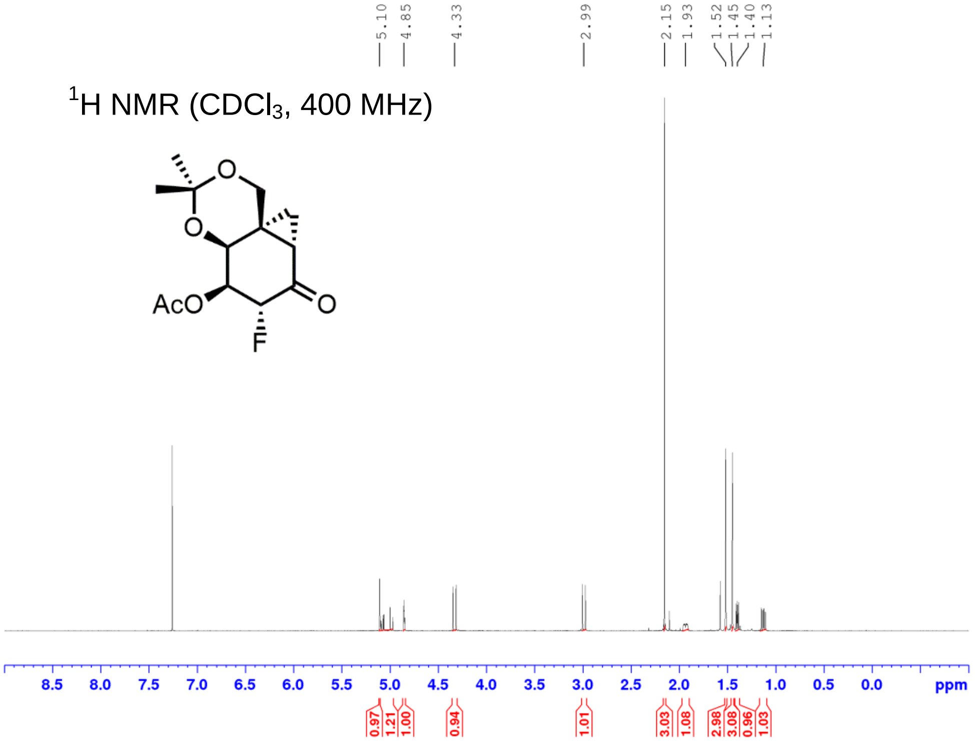
^{1}H NMR ($CDCl_3$, 400 MHz)

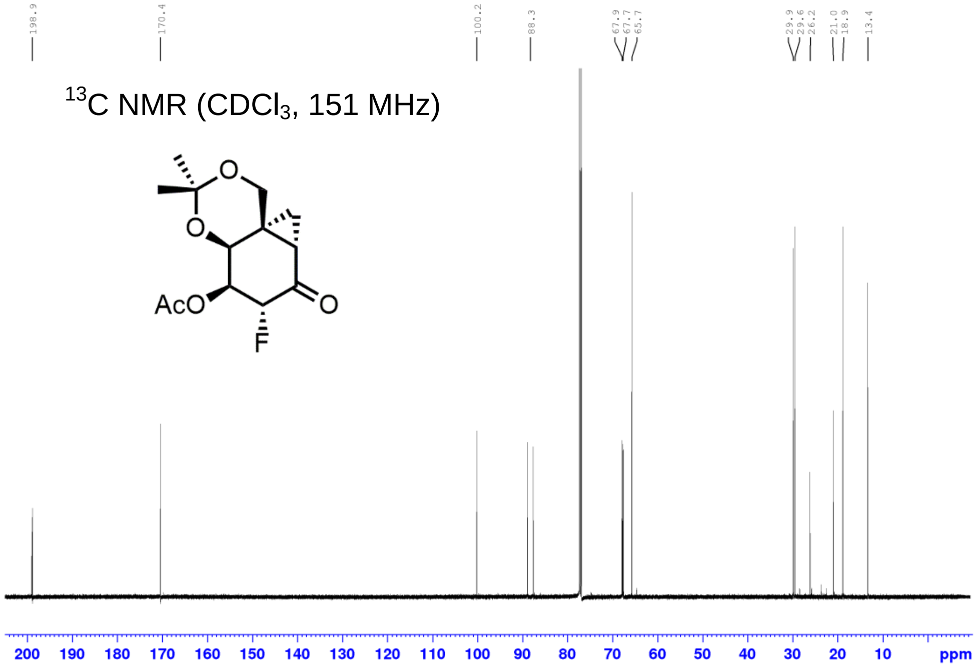
^{13}C NMR ($CDCl_3$, 151 MHz)

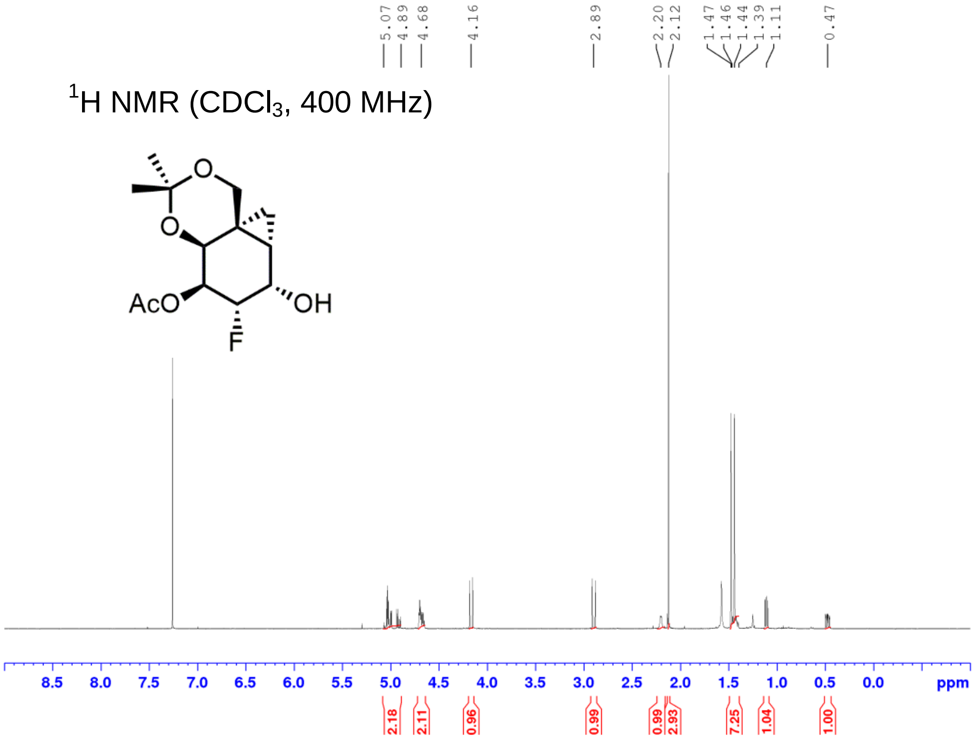
5.07
4.89
4.68
4.16
2.89
2.20
2.12
1.47
1.46
1.44
1.39
1.11
0.47
^{1}H NMR ($CDCl_3$, 400 MHz)
AcO
F
OH
O
O
8.5
8.0
7.5
7.0
6.5
6.0
5.5
5.0
4.5
4.0
3.5
3.0
2.5
2.0
1.5
1.0
0.5
0.0
ppm
2.18
2.11
0.96
0.99
0.99
2.93
7.25
1.04
1.00

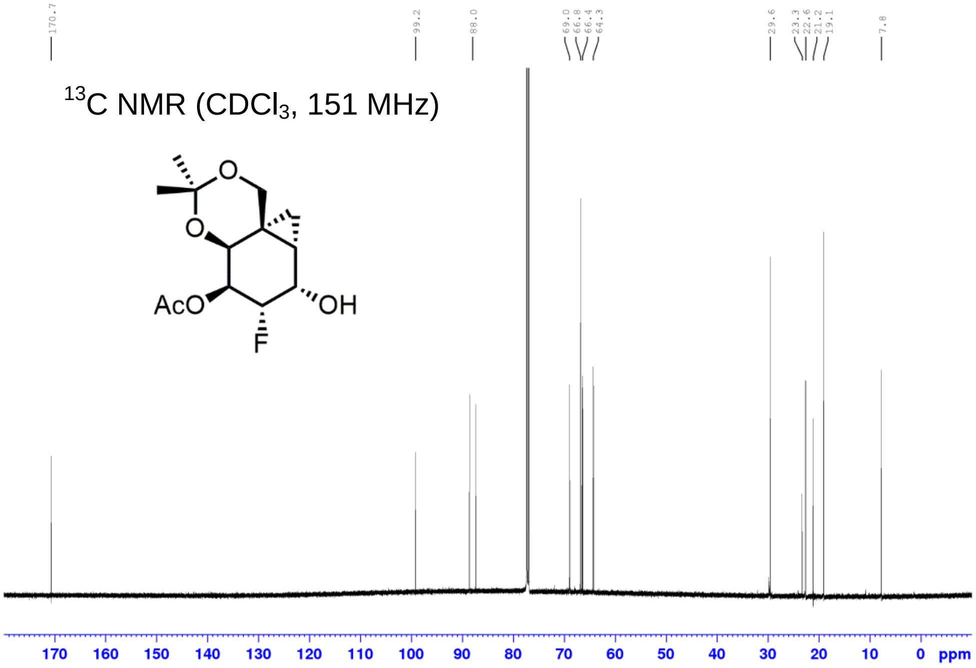
170.7
99.2
88.0
69.0
66.8
66.4
64.3
29.6
23.3
22.6
21.2
19.1
7.8
^{13}C NMR ($CDCl_3$, 151 MHz)
AcO
F
OH
O
O
170
160
150
140
130
120
110
100
90
80
70
60
50
40
30
20
10
0
ppm

^{1}H NMR ($CDCl_3$, 400 MHz)

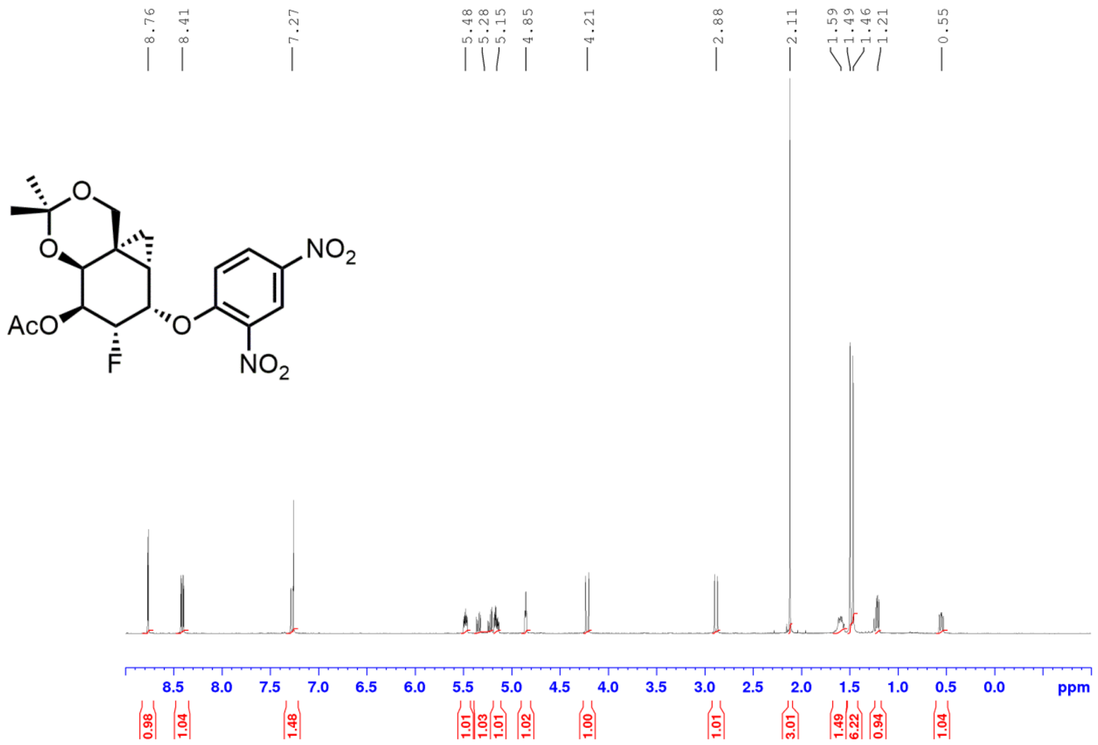

^{13}C NMR ($CDCl_3$, 151 MHz)

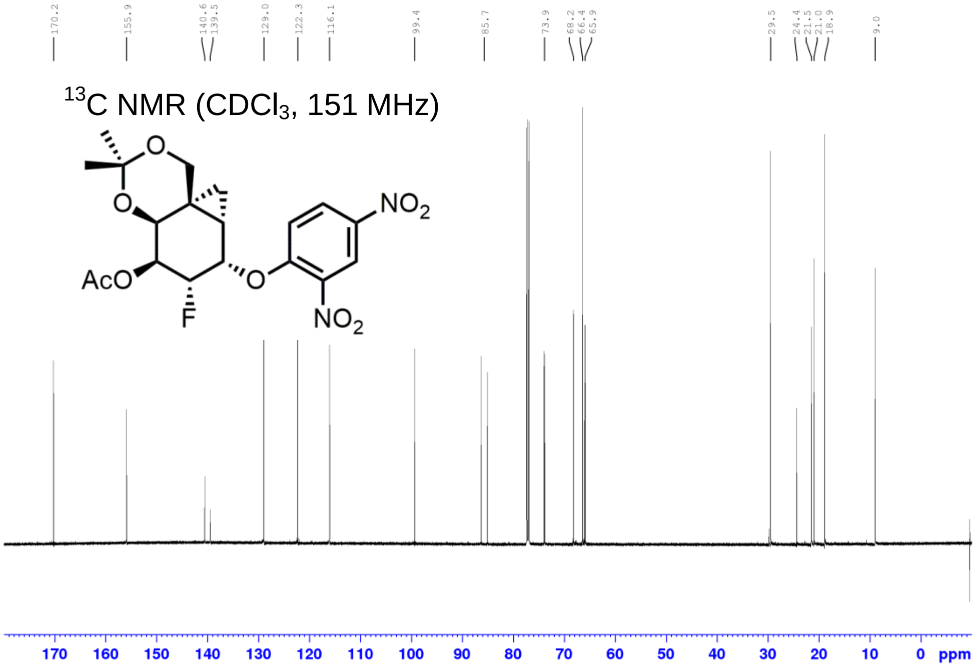

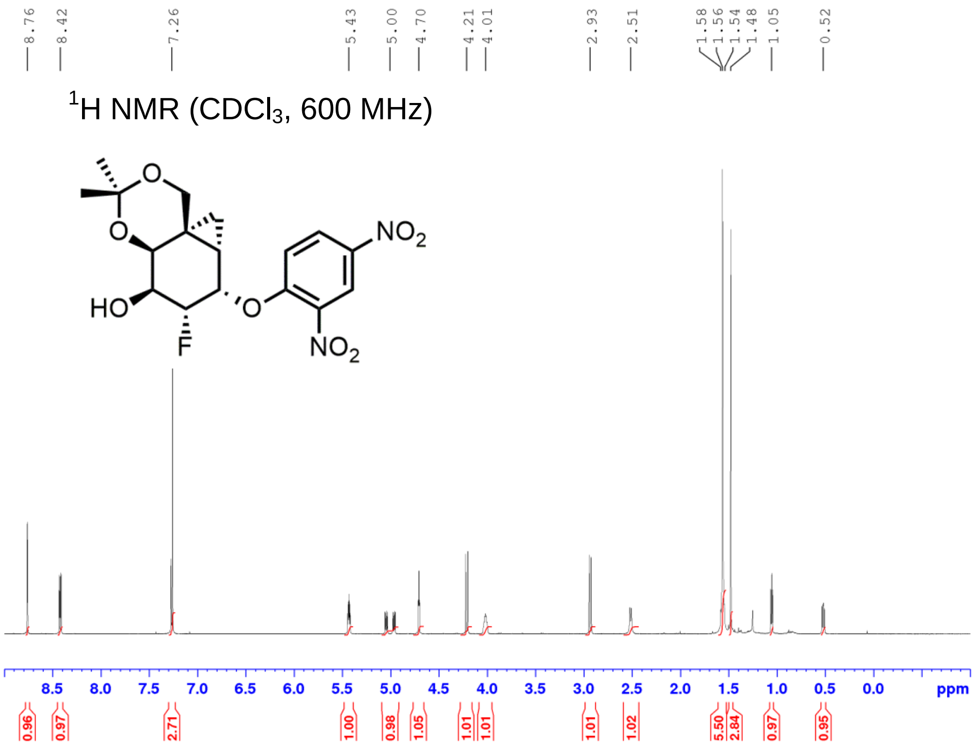
8.76
8.42
7.26
5.43
5.00
4.70
4.21
4.01
2.93
2.51
1.58
1.56
1.54
1.48
1.05
0.52
1H NMR (CDCl3, 600 MHz)
O
O
HO
F
O
NO2
NO2
8.5 8.0 7.5 7.0 6.5 6.0 5.5 5.0 4.5 4.0 3.5 3.0 2.5 2.0 1.5 1.0 0.5 0.0 ppm
0.96
0.97
2.71
1.00
0.98
1.05
1.01
1.01
1.01
1.02
5.50
2.84
0.97
0.95

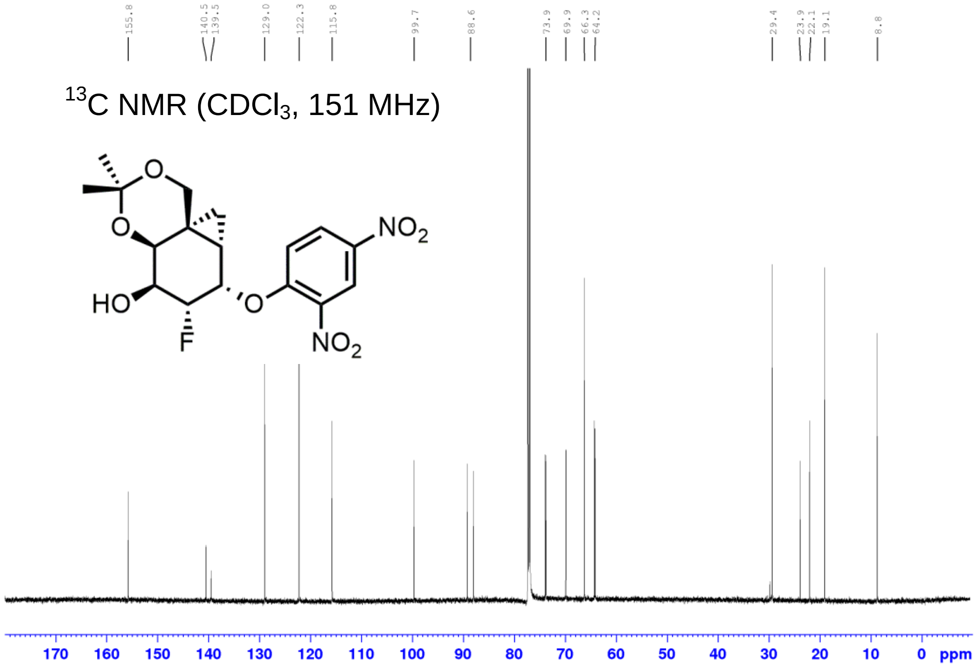
155.8
140.5
139.5
129.0
122.3
115.8
99.7
88.6
73.9
69.9
66.3
64.2
29.4
23.9
22.1
19.1
8.8
13C NMR (CDCl3, 151 MHz)
O
O
HO
F
O
NO2
NO2
170 160 150 140 130 120 110 100 90 80 70 60 50 40 30 20 10 0 ppm

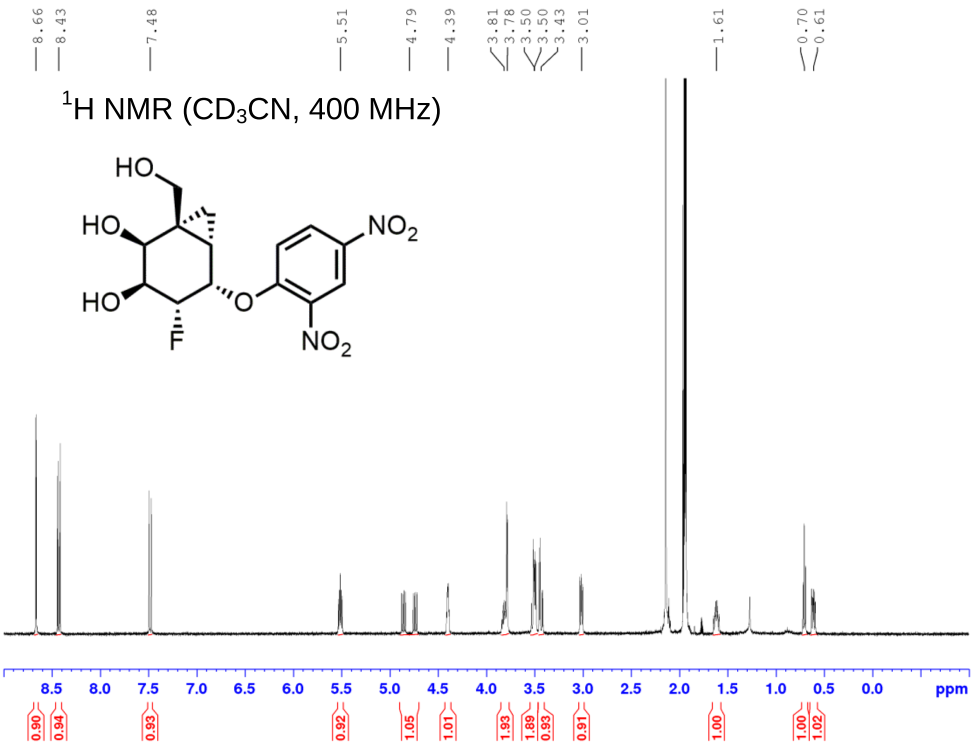

^{1}H NMR (CD_3CN, 400 MHz)

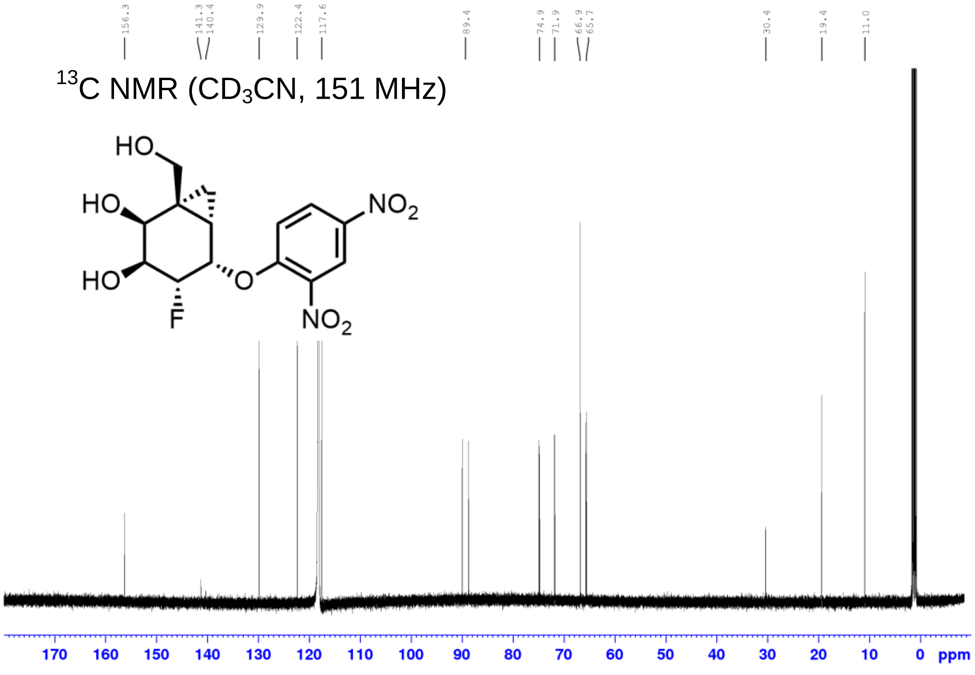

^{13}C NMR (CD_3CN, 151 MHz)
